# Innovative Air Cathode with Ni‐Doped Cobalt Sulfide in Highly Ordered Macroporous Carbon Matrix for Rechargeable Zn–Air Battery

**DOI:** 10.1002/advs.202407915

**Published:** 2024-10-14

**Authors:** Yujin Son, Kyeongseok Min, Sungkyun Cheong, Boyoung Lee, Sang Eun Shim, Sung‐Hyeon Baeck

**Affiliations:** ^1^ Department of Chemistry and Chemical Engineering Education and Research Center for Smart Energy Materials and Process Inha University Incheon 22212 Republic of Korea

**Keywords:** electrocatalysts, heteroatom dopings, inverse opal structures, transition metal sulfides, Zn–Air battery

## Abstract

To realize the practical application of rechargeable Zn–Air batteries (ZABs), it is imperative to develop a non‐noble metal‐based electrocatalyst with high electrochemical performance for the oxygen reduction reaction (ORR) and oxygen evolution reaction (OER). Herein, Ni‐doped Co_9_S_8_ nanoparticles dispersed on an inverse opal‐structured N, S co‐doped carbon matrix (IO─Ni_x_Co_9‐x_S_8_@NSC) as a bifunctional electrocatalyst is presented. The unique 3D porous structure, arranged in an inverse opal pattern, provides a large active surface area. Also, the conductive carbon substrate ensures the homogeneous dispersion of Ni_x_Co_9‐x_S_8_ nanocrystals, preventing aggregation and increasing the exposure of active sites. The introduction of heteroatom dopants into the Co_9_S_8_ structure generates defect sites and enhances surface polarity, thereby improving electrocatalytic performance in alkaline solutions. Consequently, the IO─Ni_x_Co_9‐x_S_8_@NSC shows excellent bifunctional activity with a high half‐wave potential of 0.926 V for ORR and a low overpotential of 289 mV at 10 mA cm^−2^ for OER. Moreover, the rechargeable ZAB assembled with prepared electrocatalyst exhibits a higher specific capacity (768 mAh g_Zn_^−1^), peak power density (180.2 mW cm^−2^), and outstanding stability (over 160 h) compared to precious metal‐based electrocatalyst.

## Graphical abstract

The Ni‐doped Co_9_S_8_ nanoparticles dispersed on an inverse opal‐structured N, S co‐doped carbon matrix (IO─Ni_x_Co_9‐x_S_8_@NSC) are rationally designed using a self‐sacrificial template strategy. The prepared electrocatalyst is employed as an efficient bifunctional electrocatalyst for rechargeable Zn–air batteries, exhibiting superior specific capacity, power density, and long‐term cyclability compared with the battery assembled with a precious metal‐based electrocatalyst.

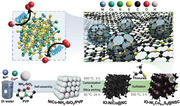

## 1 Introduction

Continuous exhaustion of fossil fuels and ever‐increasing environmental pollution have aroused an urgent request for the development of clean energy storage and conversion systems.^[^ ^1^, ^2^ ^]^ Rechargeable Zn–Air batteries (ZABs) have been regarded as one of the most promising devices to address the aforementioned challenges.^[^ ^3^, ^4^, ^5^ ^]^ ZABs offer attractive merits, including low cost, high theoretical energy density (1086 Wh kg^−1^), environmental friendliness, and inherent safety.^[^ ^6^, ^7^ ^]^ Nevertheless, the practical utilization of ZABs is severely limited due to the intrinsically sluggish kinetics of oxygen evolution reaction (OER) and oxygen reduction reaction (ORR) at the air cathode.^[^ ^8^ ^]^ To improve the electrocatalytic activities at the air cathode of ZABs, many researchers conventionally employed noble metal‐based electrocatalysts, such as Pt/C and RuO_2_ for ORR and OER, respectively.^[^ ^9^, ^10^ ^]^ Despite their high activities for oxygen‐related reactions, the development of alternative electrocatalysts is essential due to the high cost and poor electrochemical stability of noble metals.^[^ ^11^ ^]^ In this respect, transition metal‐based materials, such as oxides,^[^ ^12^ ^]^ sulfides,^[^ ^13^ ^]^ carbides,^[^ ^14^ ^]^ hydroxides,^[^ ^15^ ^]^ and phosphides^[^ ^16^ ^]^ have attracted great attention as bifunctional oxygen electrocatalysts owing to abundance in the Earth and various valence states of metal species.

Among the various transition metal‐based materials, transition metal sulfides (TMS) offer multiple benefits for electrocatalysis, such as a narrow band gap induced by the S 3p orbital, high electrical conductivity, and outstanding chemical stability in aqueous electrolytes.^[^ ^17^ ^]^ Especially, the pentlandite‐structured cobalt sulfide (Co_9_S_8_) has unique physicochemical and electronic properties. First of all, the Co_9_S_8_ typically crystallizes in a cubic crystal structure, which can expose numerous active sites available for electrocatalysis.^[^ ^18^ ^]^ Additionally, the abundant unpaired electrons in the Co d orbitals can readily interact with the oxygen species.^[^ ^19^ ^]^ The higher density of electronic states of the Co_9_S_8_ compared to other cobalt sulfide species also enhances the availability of electrons during reactions, resulting in high electrocatalytic efficiency for both ORR and OER.^[^ ^20^ ^]^

To further enhance electrocatalytic performance, introducing heteroatom dopants into the Co_9_S_8_ structure induces lattice distortion and generates numerous defect sites, which are highly beneficial for creating more efficient active sites for alkaline ORR and OER.^[^ ^21^ ^]^ Notably, the partial substitution of cobalt with foreign metal species effectively increases surface polarity and charge transfer rates by modulating the electronic structure around the active sites.^[^ ^22^, ^23^, ^24^ ^]^ Consequently, the cation doping strategy in Co_9_S_8_ significantly accelerates the inherently sluggish kinetics of oxygen‐related electrocatalysis.^[^ ^25^ ^]^ In a recent study, Qian et al.^[^ ^26^ ^]^ demonstrated, using QSTEM atomic arrangement simulations, that cation doping in the Co_9_S_8_ structure induces substantial lattice distortion and concentrates high strain levels. Nickel and cobalt‐based bimetallic sulfides have been shown to enhance bifunctional electrocatalytic activity for both ORR and OER. For example, Liu et al.^[^ ^27^ ^]^ performed DFT calculations on Ni and Co bimetallic sulfides to investigate the distinct catalytic behaviors of Ni and Co active sites. The results demonstrated that the Ni site has a lower Δ*G* value for the rate‐determining step (RDS) of OER (OOH*→O_2_) compared to the Co site. Conversely, during alkaline ORR, the Co site exhibited a lower RDS energy (OOH*→O*) than the Ni site. These findings indicate that OER and ORR are preferentially facilitated at Ni and Co active sites, respectively. Therefore, the utilization of Ni, Co‐based bimetallic sulfides, rather than monometallic sulfides, is an effective strategy for achieving bifunctionality in both OER and ORR.

The combination of a conductive carbon substrate with the active TMS can be adopted as an effective strategy for enhancing electrocatalytic activity due to its mechanical flexibility, large active surface area, and excellent electrical conductivity.^[^ ^28^ ^]^ Additionally, the active species can be homogeneously dispersed and chemically stabilized on the carbon framework, providing high corrosion resistance during the long‐term operation of ORR and OER in alkaline solution.^[^ ^29^ ^]^ To significantly enhance the geometrical surface area of the electrocatalyst, a 3D‐ordered macroporous (3DOM) structure could be employed in the preparation of the carbon framework.^[^ ^30^ ^]^ In particular, the inverse opal (IO) structure has a high specific surface area, large pore volume, and mechanically stable architecture.^[^ ^31^, ^32^ ^]^ This unique structure not only improves the mass transfer efficiency but also increases the accessibility of active sites, thereby significantly enhancing overall electrocatalytic performance for both ORR and OER.^[^ ^33^ ^]^ For example, Zhao et al.^[^ ^34^ ^]^ synthesized heterostructured Co/VN supported by N‐doped carbon (NC) with a 3DOM architecture as ORR/OER bifunctional electrocatalyst. The IO structure, designed using a polystyrene (PS) sphere template, provided interconnected channels that expedite ion/electron transportation, benefiting the electrochemical performance of rechargeable ZAB in aqueous solution.

Based on the above considerations, we propose Ni‐doped Co_9_S_8_ nanoparticles dispersed on an IO‐structured N, S co‐doped carbon matrix (IO─Ni_x_Co_9‐x_S_8_@NSC) as an efficient bifunctional electrocatalyst for ORR, OER, and rechargeable ZAB. The proposed electrocatalyst was synthesized via self‐assembly, annealing, template etching, and sulfidation process. We employed silica spheres as a self‐sacrificial template, and the surface‐functionalized silica spheres were stacked throughout the self‐assembly procedure, resulting in a uniform‐sized inverse opal structure after the template removal. Through an elaborate synthetic procedure, Ni dopants are incorporated into the Co_9_S_8_ structure, while N and S dopants are simultaneously dispersed onto the carbon substrate. The Ni dopants enhance the intrinsic redox properties of Co_9_S_8_ by enabling the active sites to adopt multiple valence states.^[^ ^35^ ^]^ Additionally, the heteroatom dopants within the carbon matrix effectively disrupt the electro‐neutrality of the sp^2^ carbon structure and modify the electron spin density, thereby improving surface polarity and charge transfer efficiency during electrocatalytic reactions.^[^ ^36^ ^]^ Consequently, the prepared IO─Ni_x_Co_9‐x_S_8_@NSC electrocatalyst exhibited outstanding electrocatalytic performance for both ORR and OER in an alkaline solution. Also, rechargeable ZAB using IO─Ni_x_Co_9‐x_S_8_@NSC as the air cathode achieved superior specific capacity, high peak power density, and long‐term durability compared with a ZAB assembled with precious metal‐based electrocatalysts.

## 2 Results and Discussion

### 2.1 Synthetic Procedure of IO─Ni_x_Co_9‐x_S_8_@NSC

**Scheme 1:**
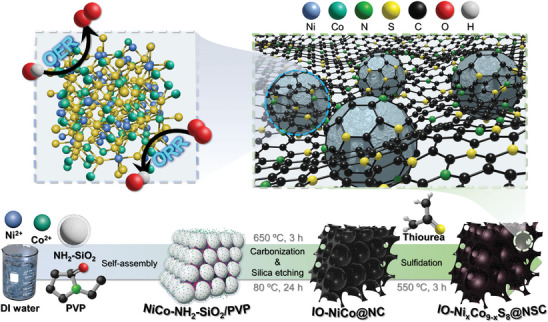
Schematic illustration of IO─Ni_x_Co_9‐x_S_8_@NSC synthesis strategy.

The synthesis procedure of Ni_x_Co_9‐x_S_8_ nanoparticles dispersed on an N, S co‐doped carbon matrix with an inverse opal structure is schematically illustrated in **Scheme** **1**. First, amine‐functionalized silica (NH_2_‐SiO_2_) nanospheres were fabricated by the well‐known Stöber method through the ammonia‐catalyzed reaction of tetraethyl orthosilicate (TEOS), followed by surface treatment with 3‐aminopropyltrimethoxysilane (APTMS) at 80 °C. During the surface functionalization process, the ─OH terminal groups of SiO_2_ sphere were partially replaced by the ─NH_2_ groups, which attract metal ions more effectively than hydroxyl groups.^[^ ^37^ ^]^ Subsequently, the NH_2_‐SiO_2_ nanospheres were homogeneously dispersed in DI water, followed by the adsorption of Ni and Co ions. During this process, the metal ions easily coordinated with the N atoms on the amino‐terminated SiO_2_ surface owing to the electrostatic interaction.^[^ ^37^ ^]^ After that, the metal ion‐coordinated NH_2_‐SiO_2_ spheres formed a closely packed opal template via vacuum filtration. Simultaneously, the PVP species tightly filled the void interspaces between the opal templates, resulting in a dense filter cake denoted as “NiCo‐NH_2_‐SiO_2_/PVP”. The filter cake was dried in a vacuum oven at 60 °C overnight and ground into a powder. The powdery NiCo‐NH_2_‐SiO_2_/PVP sample was carbonized at 650 °C for 3 h under Ar flow, followed by a silica etching process with 2 m NaOH solution. In this process, the PVP filling the interspace between opal templates transformed into the conductive N‐doped carbon framework. Meanwhile, the SiO_2_ was completely etched out by the harsh alkaline solution, constructing a uniform inverse opal structured N‐doped carbon framework decorated by NiCo alloy nanoparticles (IO─NiCo@NC). Finally, the IO─NiCo@NC was thermally sulfurized using thiourea in the tube furnace at 550 °C for 3 h under an Ar atmosphere. The highly active H_2_S gases are generated from the thermal decomposition of thiourea at high temperatures, thereby converting metal species to sulfides and simultaneously doping the carbon with sulfur.^[^ ^38^ ^]^ The nickel dopants are homogeneously introduced to pentlandite‐structured cobalt sulfide (Co_9_S_8_), which causes lattice distortion and generates numerous active sites for alkaline ORR and OER. Additionally, the N, S dual‐doped carbon framework with an inverse opal structure not only provides a large specific surface area but also improves the electrical conductivity of the prepared electrocatalyst.

### 2.2 Physicochemical Characterization of Prepared Electrocatalysts

First, we compared the crystal structures and surface morphologies of the as‐prepared SiO_2_ and NH_2_‐SiO_2_ using X‐ray diffraction (XRD) and scanning electron microscopy (SEM) measurements (Figure S1, Supporting Information). In the XRD patterns, a broad diffraction peak at ≈22° is observed for both SiO_2_ and NH_2_‐SiO_2_ samples, signifying the presence of amorphous SiO_2_.^[^ ^39^ ^]^ Also, the particle size and surface morphology of SiO_2_ are almost identical before and after amine‐functionalization, as shown in the SEM images. Fourier‐transform infrared (FT‐IR) measurements were conducted on the SiO_2_ and NH_2_‐SiO_2_ samples, as shown in Figure S2, Supporting Information. In the FT‐IR spectrum of pristine SiO_2_, the bending and stretching vibrations of ─OH are observed at 1628 and 3427cm^−1^, respectively.^[^ ^40^ ^]^ Additionally, characteristic peaks located at 808 and 1103 cm^−1^ are associated with the Si─O─Si symmetric and asymmetric stretching vibrations, respectively.^[^ ^40^ ^]^ Meanwhile, additional peaks appear in the NH_2_‐SiO_2_ sample at 1405 and 3150 cm^−1^, manifesting the bending and stretching vibration of amine groups, respectively.^[^ ^40^ ^]^ The surface charge property was altered after amine‐functionalization, as confirmed in the zeta potential measurement (Figure S3, Supporting Information). The NH_2_‐SiO_2_ exhibits a zeta potential value of −32.60 mV, which is more positive than pristine SiO_2_ (−44.47 mV).^[^ ^41^ ^]^ Based on the above results, it could be concluded that the surface of the SiO_2_ sphere was successfully functionalized by the amine group.

**Figure 1:**
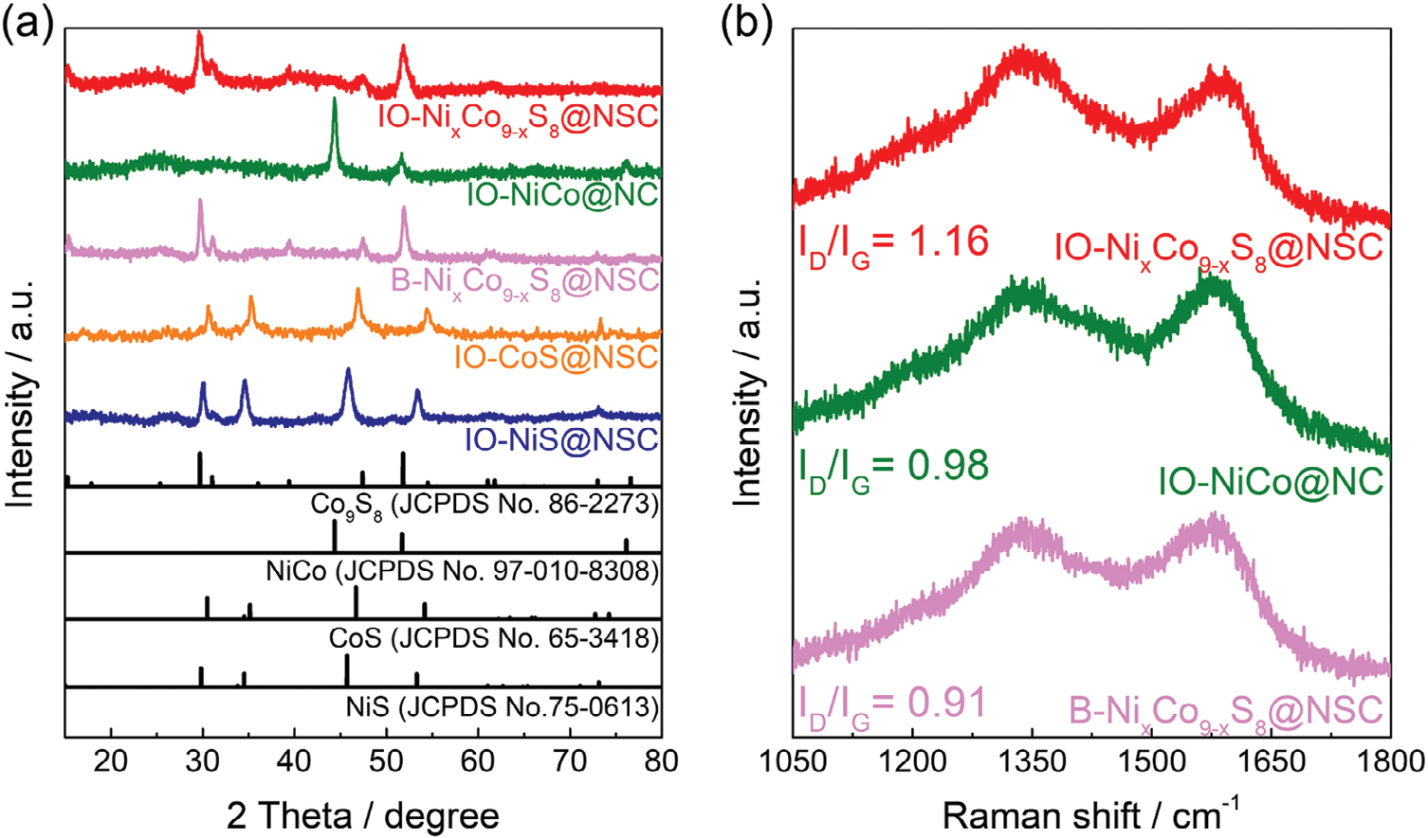
a) X‐ray diffraction patterns as‐prepared all samples. b) Raman spectra of IO─Ni_x_Co_9‐x_S_8_@NSC, IO─NiCo@NC, and B‐Ni_x_Co_9‐x_S_8_@NSC.

After the carbonization and etching process using the NiCo‐NH_2_‐SiO_2_/PVP, the XRD patterns of IO─NiCo@NC revealed three significant diffraction peaks at 44.4°,51.8° and 76.3°, which originated from (111), (200) and (220) lattice planes of the face‐centered‐cubic (fcc) NiCo alloy, respectively (**Figure** **1**a).^[^ ^42^ ^]^ In comparison to the XRD pattern of NiCo‐NH_2_‐SiO_2_@NC (before silica etching), the broad characteristic peak for SiO_2_ disappeared in the XRD pattern of IO─NiCo@NC, demonstrating that the SiO_2_ template was completely etched out by alkaline solution (Figure S4, Supporting Information). After the sulfidation of IO─NiCo@NC, the XRD pattern of IO─Ni_x_Co_9‐x_S_8_@NSC exhibited five apparent major peaks at 15.4°, 29.8°, 31.2°, 47.4°, and 51.8°, corresponding to the (111), (311), (222), (511), and (440) lattice planes of cubic‐structured Co_9_S_8_ (JCPDS No. 86–2273).^[^ ^43^ ^]^ There are no characteristic peaks for NiCo alloy, indicating the complete sulfidation of metal species without the formation of other impurities. Also, the B‐Ni_x_Co_9‐x_S_8_@NSC prepared without inverse opal structure (in the absence of SiO_2_ template) exhibited an identical crystallographic nature to the IO─Ni_x_Co_9‐x_S_8_@NSC. Interestingly, monometallic control samples prepared with the same synthetic procedure of the IO─Ni_x_Co_9‐x_S_8_@NSC showed hexagonal NiS and CoS crystal structures (JCPDS No. 75–0613 and 65–3418, respectively).^[^ ^44^, ^45^ ^]^ This result shows that the use of dual‐metal species induced the lattice distortion and phase transformation, resulting in the formation of highly active pentlandite‐structured metal sulfide. Raman spectroscopy was also performed to analyze the chemical structure of carbonaceous species in the as‐prepared samples.^[^ ^46^ ^]^ In Figure 1b, two intense and broad peaks located at 1580 and 1340 cm^−1^ are ascribed to the D and G bands of carbon, respectively.^[^ ^47^ ^]^ The D band corresponds to the lattice defect and sp^3^ hybridized carbon, whereas the G band signifies graphitic sp^2^ hybridized carbon. In general, the intensity ratio of the D band to the G band (I_D_/I_G_) indicates the degree of carbon defects such as vacancies, lattice mismatches, edge sites, and heteroatom doping.^[^ ^48^, ^49^ ^]^ In this study, the I_D_/I_G_ values for IO─Ni_x_Co_9‐x_S_8_@NSC, IO─NiCo@NC, B‐Ni_x_Co_9‐x_S_8_@NSC were calculated to be 1.16, 0.98, and 0.91, respectively. The highest *I*_D_/*I*_G_ value of IO─Ni_x_Co_9‐x_S_8_@NSC is attributed to the introduction of N, S dual‐dopants and topological defects induced by the porous inverse opal structure.^[^ ^50^ ^]^ Such abundant structural defects within the carbon matrix promote alterations in the electronic configuration.^[^ ^51^ ^]^ Therefore, electrochemically unstable sites serve as effective active sites for both ORR and OER in alkaline media.^[^ ^52^ ^]^

**Figure 2:**
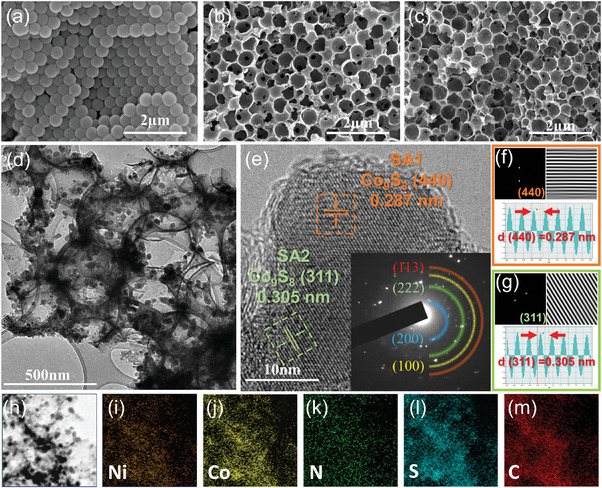
SEM images of a) NiCo‐NH_2_‐SiO_2_@NC, b) IO─NiCo@NC and c) IO─Ni_x_Co_9‐x_S_8_@NSC. d) Low‐magnification TEM image of IO─Ni_x_Co_9‐x_S_8_@NSC, e) HRTEM image of IO─Ni_x_Co_9‐x_S_8_@NSC with the index of Co_9_S_8_ plane; the inset shows the SAED patterns of IO─Ni_x_Co_9‐x_S_8_@NSC. FFT images, IFFT patterns, and contrast intensity profiles correspond to f) SA1 and g) SA2. h–m) Elemental mapping images of IO─Ni_x_Co_9‐x_S_8_@NSC.

**Figure 3:**
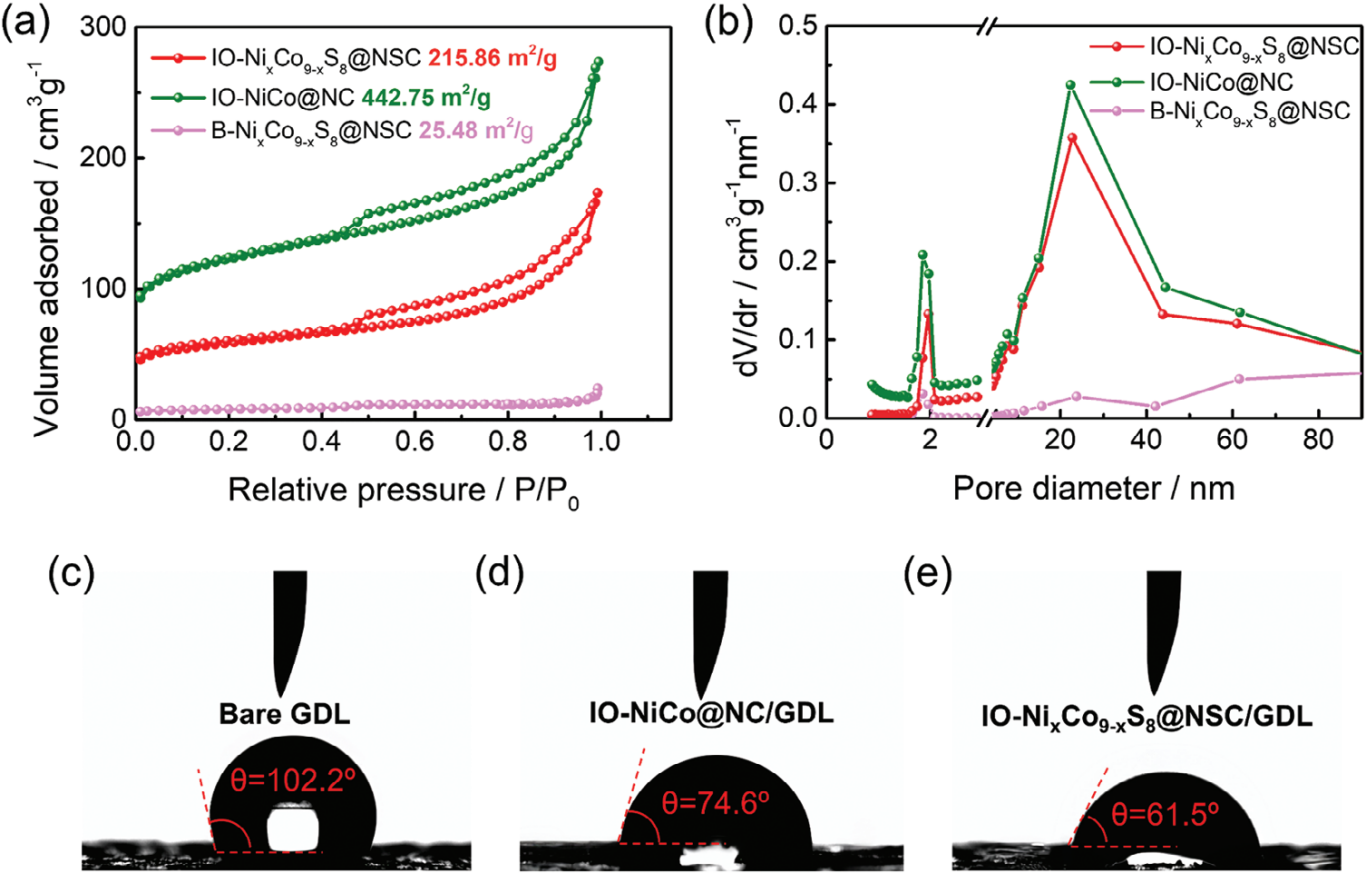
a) N_2_ adsorption‐desorption isotherms and b) pore‐size distributions of IO─Ni_x_Co_9‐x_S_8_@NSC, IO─NiCo@NC, and B‐NixCo_9‐x_S_8_@NSC. Contact angle images of water droplets on the surface of c) bare GDL, d) IO─NiCo@NC/GDL, and e) IO─Ni_x_Co_9‐x_S_8_@NSC/GDL.

To investigate the surface morphologies and interior nanostructures, scanning electron microscopy (SEM) and transmission electron microscopy (TEM) measurements were conducted using the prepared samples. First, the SEM image of NiCo‐NH_2_‐SiO_2_/PVP shows the uniformly ordered opal structure based on the self‐assembly process (Figure S5, Supporting Information). Even after the carbonization process at 650 °C, the highly ordered structure was well‐maintained, as shown in the SEM image of NiCo‐NH_2_‐SiO_2_@NC (**Figure** **2**a). After the silica etching and sulfidation processes, the 3D‐ordered inverse opal structure was well‐defined with interconnected meso/macropores (Figure 2b,c). The unique inverse opal structure increases the active surface area of the electrocatalyst, consequently enhancing its electrocatalytic performance for both ORR and OER. In the TEM image of IO─Ni_x_Co_9‐x_S_8_@NSC, the Ni_x_Co_9‐x_S_8_ nanoparticles are homogeneously dispersed on the inverse opal‐structured NSC matrix (Figure 2d). In contrast, the B‐Ni_x_Co_9‐x_S_8_@NSC showed a highly aggregated morphology with no distinct ordered structure (Figure S6, Supporting Information). Additionally, the thick and dense carbon matrix impeded homogeneous heat transfer, resulting in uneven growth of the sulfide nanocrystals.^[^ ^53^, ^54^ ^]^ In the high‐resolution TEM (HRTEM) image, each nanoparticle in the IO─Ni_x_Co_9‐x_S_8_@NSC was scrutinized (Figure 2e). The lattice spacings of 0.287 and 0.305 nm were observed in the HRTEM image, which is attributed to the (440) and (311) planes of cubic‐structured Co_9_S_8_, respectively. Additionally, the inset of **Figure** **3**e represents the selected area electron diffraction (SAED) pattern. In the SAED pattern, several diffraction rings manifest the polycrystalline nature of the Co_9_S_8_ particles, signifying the (200), (222), (100), and (113) lattice planes of Co_9_S_8_, which is in accordance with the above XRD results. Moreover, Figure 3f,g displays a fast Fourier transform (FFT) pattern, inverse FFT (IFFT) image, and contrast intensity profile corresponding to the selected regions in Figure 2e (marked by orange and green dashed lines). The elemental distribution of IO─Ni_x_Co_9‐x_S_8_@NSC was confirmed through energy‐dispersive spectroscopy (EDS) elemental mapping (Figure 3h–m). The elemental composition determined by EDS analysis was Ni:Co:N:S:C = 2.21:3.70:15.81:7.17:71.11 (Table S1, Supporting Information). Additionally, the elemental content of IO─Ni_x_Co_9‐x_S_8_@NSC was further explored by inductively coupled plasma optical emission spectrometry (ICP‐OES) and elemental analysis (EA), as summarized in Table S1, Supporting Information. The atomic ratios of the constituent elements obtained from ICP‐OES and EA measurements are in good agreement with the EDS results.

The specific surface area and pore‐size distribution of the prepared electrocatalysts were determined by the Brunauer‐Emmett‐Teller (BET) method using nitrogen ad/desorption measurements (Figure 3a,b). First, the isotherm curves of IO─Ni_x_Co_9‐x_S_8_@NSC and IO─NiCo@NC showed the type IV isotherm curves with distinct hysteresis loops, indicating the presence of a mesoporous structure.^[^ ^55^ ^]^ Meanwhile, the B‐Ni_x_Co_9‐x_S_8_@NSC sample exhibited a lower degree of nitrogen adsorption, resulting in a smaller specific surface area of 25.48 m^2^ g^−1^ compared to IO─Ni_x_Co_9‐x_S_8_@NSC (215.86 m^2^ g^−1^) and IO─NiCo@NC (442.75 m^2^ g^−1^). Interestingly, the surface area of IO─Ni_x_Co_9‐x_S_8_@NSC was decreased after the sulfidation, which may be ascribed to self‐aggregation and partial collapse of porous structure during thermal treatment.^[^ ^56^ ^]^ As expected, the total pore volume of IO─NiCo@NC (0.322 cm^3^ g^−1^) was higher than that of IO─Ni_x_Co_9‐x_S_8_@NSC (0.304cm^3^ g^−1^) (Figure 3b). Notably, the well‐defined IO structure samples exhibited a larger surface area and developed pore size distributions compared to the bulk structure sample. The existence of meso/macropores in the IO‐structured electrocatalysts can provide the mass transport channels for electrolytes and involved gases, thereby contributing to the enhancement of rechargeable ZAB performance.^[^ ^57^ ^]^ In addition, the surface polarity and hydrophilicity of the catalyst layer significantly affect the electrocatalytic activity in aqueous conditions.^[^ ^58^ ^]^ During the electrochemical reaction of ZAB, a hydrophilic electrocatalyst can effectively mitigate concentration polarization caused by mass transfer, thereby improving the charge–discharge efficiency of ZAB.^[^ ^59^ ^]^ As shown in Figure 3c–e, the wettability test indicated that the IO─Ni_x_Co_9‐x_S_8_@NSC‐coated GDL has a substantially lower contact angle (61.5°) than IO─NiCo@NC‐coated GDL (74.6°) and bare GDL (102.2°). These results indicate that the introduction of sulfur into IO─Ni_x_Co_9‐x_S_8_@NSC increases surface polarity, thereby facilitating improved interactions between reactants and active sites on the catalyst layer during electrocatalysis.^[^ ^36^ ^]^

**Figure 4:**
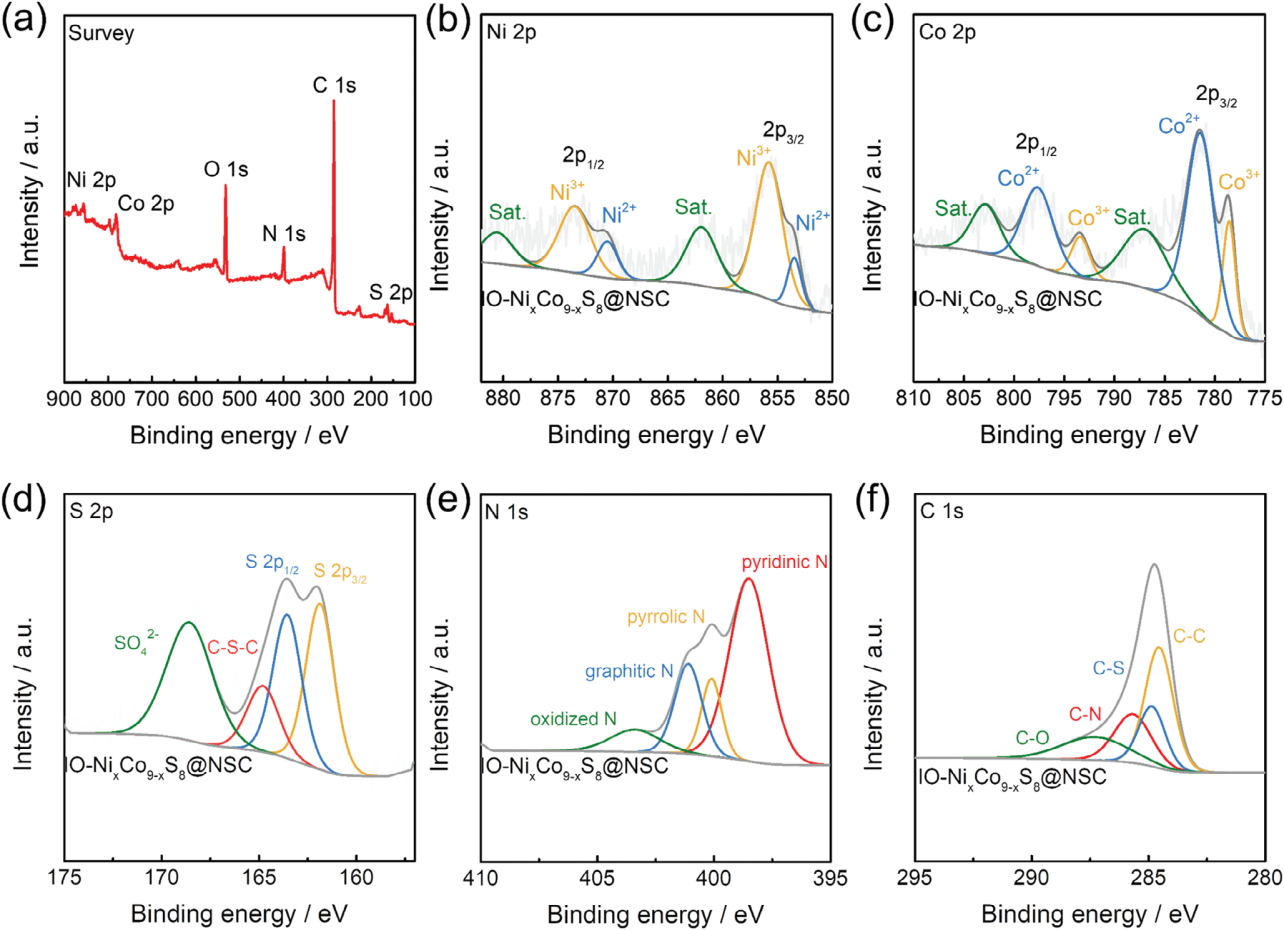
a) Full scan XPS survey spectrum of IO─Ni_x_Co_9‐x_S_8_@NSC and High‐resolution XPS spectra of b) Ni 2p, c) Co 2p, d) S 2p, e) N 1s, and f) C 1s for the IO─Ni_x_Co_9‐x_S_8_@NSC.

The valence states and chemical composition of the IO─Ni_x_Co_9‐x_S_8_@NSC electrocatalyst were investigated by X‐ray photoelectron spectroscopy (XPS) measurements (**Figure** **4**). The survey spectrum of IO─Ni_x_Co_9‐x_S_8_@NSC confirms the presence of six elements without any impurity, which is consistent with the above EDS results. In the Ni 2p spectrum (Figure 4b), two pairs of coupled peaks emerged. The peaks at 853.3 and 870.5 eV are ascribed to the Ni 2p_3/2_ and Ni 2p_1/2_ of Ni^2+^, respectively. Similarly, the other peaks at 855.7 and 873.4 eV correspond to those of Ni^3+^, revealing the successful doping of nickel species into the cobalt sulfide structure with certain chemical bonding.^[^ ^60^ ^]^ The Co_9_S_8_ structure is composed of octahedral Co^3+^ sites and tetrahedral Co^2+^ sites. The higher intensity of Ni^3+^ compared to that of Ni^2+^ manifests that the Ni dopants are mainly introduced into octahedral sites by a partial substitution mechanism, which can enhance the electrical conductivity of the pristine Co_9_S_8_ structure.^[^ ^61^, ^62^ ^]^ Meanwhile, the high‐resolution Co 2p XPS spectrum (Figure 4c) displays four distinct peaks in both Co 2p_3/2_ and Co 2p_1/2_ regions, corresponding to Co^3+^(778.58 and 793.60 eV) and Co^2+^(781.37 and 797.32 eV).^[^ ^63^ ^]^ This observation further confirms the formation of metal‐sulfur bonding within the Co_9_S_8_ structure.^[^ ^64^, ^65^ ^]^ In contrast to the Ni 2p XPS spectrum, the peak intensity of Co^3+^ species is lower than that of Co^2+^ due to the partial substitution of Co^3+^ by the Ni dopant, which mainly exists as Ni^3+^ states. In the high‐resolution S 2p spectrum (Figure 4d), the two peaks at 161.8 and 163.5 eV are ascribed to the 2p_1/2_ and 2p_3/2_ core levels of metal‐sulfur bonding.^[^ ^66^ ^]^ Additionally, the other peaks at 164.8 and 168.5 eV correspond to carbon matrix integrated sulfur dopants and oxidized sulfate species (SO_4_^2−^).^[^ ^66^ ^]^ The formation of oxidized sulfate species is attributed to the partial oxidation of sulfur on the surface of the catalyst in ambient air conditions. The N 1s XPS spectrum is deconvoluted into four peaks signifying pyridinic‐N (398.7 eV), pyrrolic‐N (400.3 eV), graphitic‐N (401.4 eV), and oxidized‐N (403.1 eV) species (Figure 4e).^[^ ^67^ ^]^ The aforementioned S 2p and N 1s XPS spectra demonstrate the successful incorporation of sulfur and nitrogen dopants into the carbon matrix, leading to the creation of numerous defect sites.^[^ ^68^ ^]^ These defect sites enhance the adsorption of reactants during both ORR and OER in alkaline solutions.^[^ ^68^ ^]^ Typically, pyridinic N, located at the edge of the graphitic carbon structure, serves as an active site for the ORR owing to the lone‐pair electron in the sp^2^ orbital.^[^ ^69^ ^]^ Moreover, graphitic N species enhance the electrical conductivity of carbon‐based materials, consequently facilitating electron transfer during electrocatalysis.^[^ ^69^ ^]^ As summarized in Figure S9 (Supporting Information), the combined proportion of pyridinic and graphitic N increased after sulfidation (74.1%) compared to before (65.2%). This result suggests that the introduction of additional sulfur dopants influenced the nitrogen configuration in the inverse opal carbon matrix. In the high‐resolution C 1s XPS spectrum (Figure 4f), four deconvoluted peaks at 284.6, 284.9, 285.6 and 287.2 eV represent the C─C, C─S, C─N, and O─C─O bonding, respectively, further demonstrating the introduction of dual dopants in the carbon matrix.^[^ ^70^, ^71^ ^]^

### 2.3 Electrochemical ORR Performance of Prepared Electrocatalysts

**Figure 5:**
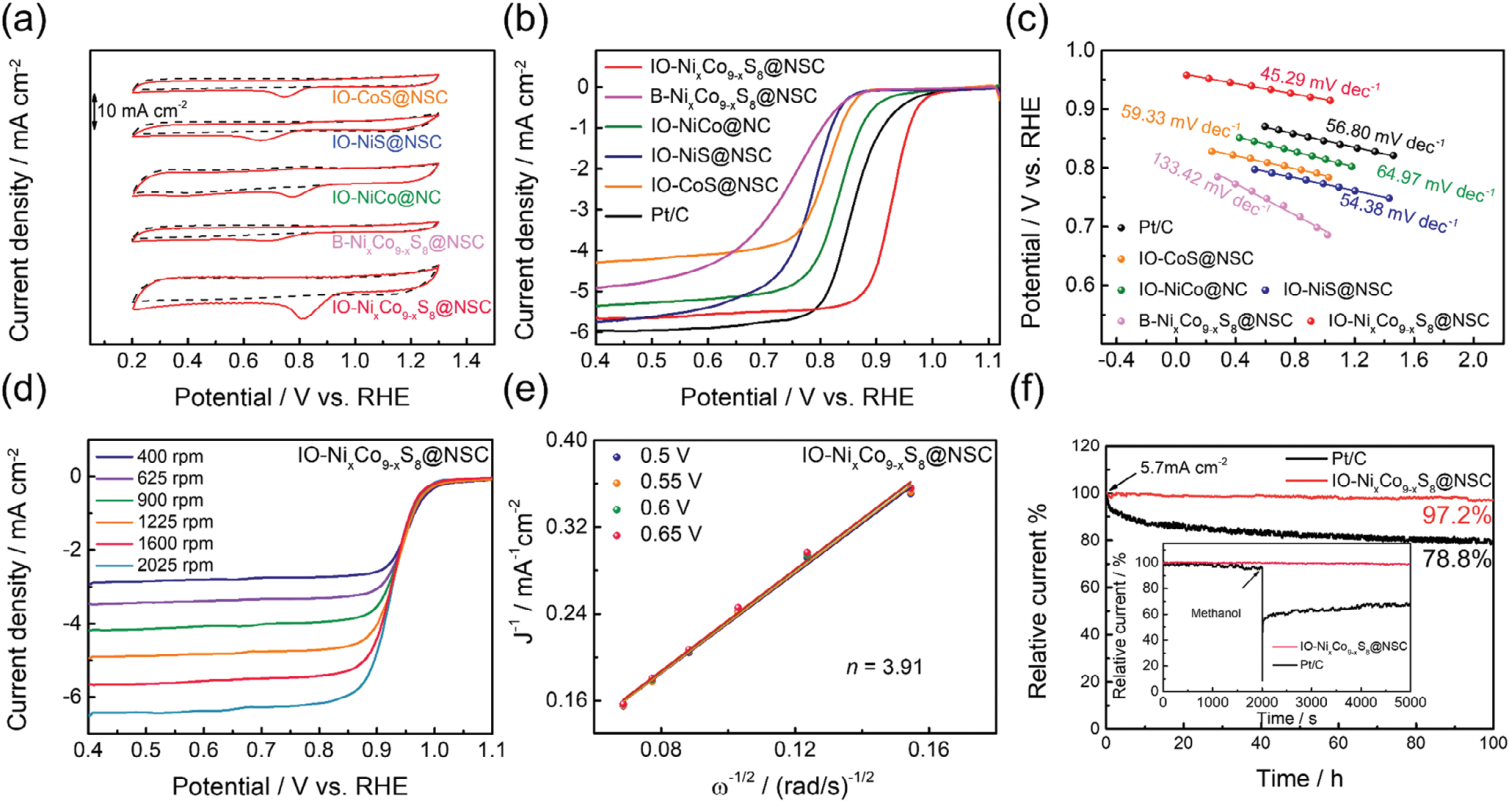
Evaluation of the ORR performances of the as‐prepared IO─Ni_x_Co_9‐x_S_8_@NSC, B‐Ni_x_Co_9‐x_S_8_@NSC, IO─NiCo@NC, IO─NiS@NSC, IO‐CoS@NSC and commercial Pt/C a) CV curves measured in N_2_‐saturated (black dash line) and O_2_‐saturated (red solid line) 0.1 m KOH solution. b) LSV polarization curves with a rotation speed of 1600 r min^−1^ c) Tafel plots. d) LSV curves of IO─Ni_x_Co_9‐x_S_8_@NSC at various rotation speeds of 400–2025 r min^−1^_._ e) the Koutecky–Levich plots at different applied potentials. f) CA response of IO─Ni_x_Co_9‐x_S_8_@NSC at a constant applied potential of 0.6 V versus RHE; the inset shows the Methanol tolerance test using CA measurements.

The electrocatalytic ORR performances of the prepared electrocatalysts were evaluated using a standard three‐electrode setup in a 0.1 m KOH solution. First, cyclic voltammetry (CV) was conducted in N_2_‐(black dash line) and O_2_‐(red solid line) saturated electrolytes within the voltage range between 0.2 and 1.3 V versus RHE (**Figure** **5**a). All CV curves were recorded without employing a rotating electrode system. Among the CV curves obtained in N_2_ conditions, the IO─Ni_x_Co_9‐x_S_8_@NSC sample exhibited the largest rectangular area, indicating the highest double‐layer capacitance. In an O_2‐_saturated electrolyte, the IO─Ni_x_Co_9‐x_S_8_@NSC exhibited a higher cathodic peak potential for ORR compared to the other control samples. Subsequently, in contrast to the CV measurement, a rotating disk electrode (RDE) was employed during the linear sweep voltammetry (LSV) test to examine the oxygen reduction reaction (ORR) activity, with a rotation rate of 1600 rpm. As shown in Figure 5b, the LSV tests reveal that the IO─Ni_x_Co_9‐x_S_8_@NSC catalyst has a more positive half‐wave potential (E_1/2_) of 0.926 V versus RHE compared to B‐Ni_x_Co_9‐x_S_8_@NSC (0.742 V versus RHE), IO─NiCo@NC (0.832 V versus RHE), IO─NiS@NSC (0.778 V versus RHE), IO‐CoS@NSC (0.808 V versus RHE), and is even superior to the state‐of‐the‐art Pt/C (0.858 V versus RHE). The Tafel slope is a crucial parameter for investigating the reaction kinetics of advanced electrocatalysts. In Figure 5c, Tafel plots are presented based on the ORR LSV polarization curves. The Tafel slope for IO─Ni_x_Co_9‐x_S_8_@NSC (45.29 mV dec^−1^) is determined to be the lowest among all other prepared samples and commercial Pt/C, suggesting significantly enhanced ORR kinetics. A comparison of electrocatalytic ORR performance with recently reported transition metal‐based electrocatalysts is provided in Table S3 (Supporting Information), which proves that the IO─Ni_x_Co_9‐x_S_8_@NSC reported here significantly surpasses most of the reported ORR electrocatalysts.

To gain deeper insight into the ORR performance of the prepared electrocatalysts, LSV polarization curves were acquired at different rotation speeds ranging from 400 to 2025 rpm (Figure 5d; Figure S10, Supporting Information). Based on the LSV curves, we obtained the Koutechy–Levich (*K*–*L*) plots at various potentials from 0.5 to 0.65 V versus RHE (Figure 5e; Figure S11). In the K‐L plots, there is a linear correlation between J^−1^ and ω^−1/2^, signifying first‐order reaction kinetics with respect to the dissolved oxygen concentration. As a result, the average electron transfer number (*n*) of IO─Ni_x_Co_9‐x_S_8_@NSC was calculated to be 3.91, which is a much higher value than those of the other control samples, indicating that the ORR with this electrocatalyst proceeds via a desirable four‐electron process. Moreover, the hydrogen peroxide yield (%HO_2_^−^) and *n* values were further evaluated using the ring‐disk electrode (RRDE) measurements (Figure S12, Supporting Information). Along with the increase of disk current density (*I_disk_*), the ring current density (*I_ring_*) gradually appeared. The calculated *n* value and %HO_2_^−^ of IO─Ni_x_Co_9‐x_S_8_@NSC were determined to be 3.90−3.93 and 5.02%−3.57%, respectively, in the potential range from 0.5 to 0.7 V versus RHE. These results are consistent with the results obtained from the K‐L plots, confirming the high product selectivity of IO─Ni_x_Co_9‐x_S_8_@NSC during the alkaline ORR.

The long‐term stability of the oxygen reduction reaction (ORR) was evaluated using both IO─Ni_x_Co_9‐x_S_8_@NSC and commercial Pt/C. Generally, the alkaline ORR activity may decrease over extended operation due to various degradation mechanisms, including a) oxidative attack by reaction intermediates, b) demetallation of the metal‐active sites, c) protonation followed by anionic adsorption, and d) micropore flooding.^[^ ^72^ ^]^ As shown in Figure 5f, the chronoamperometric (CA) measurements demonstrate that the IO─Ni_x_Co_9‐x_S_8_@NSC sample exhibits excellent long‐term stability, retaining 97.2% of its initial current density (5.7 mA cm^−2^) after continuous operation for 100 hours. This retention value is significantly higher compared to that of commercial Pt/C, which shows a retention of 78.8%. This exceptional electrocatalytic durability can be attributed to several unique characteristics of the prepared electrocatalyst. First, the Ni_x_Co_9‐x_S_8_ active species are uniformly distributed and exhibit strong interactions with the porous carbon matrix, which effectively prevents the demetallation of metal‐active sites during the stability test. Additionally, the highly ordered inverse‐opal structure provides mechanically stable meso/macropores, which helps mitigate micropore flooding. Furthermore, in the context of direct methanol fuel cell (DMFC) applications, methanol tolerance is crucial for maintaining stable fuel cell operation. Pt‐based electrocatalysts, in particular, are vulnerable to poisoning by CO‐like intermediate species generated during the methanol oxidation process, leading to a significant decline in catalytic activity.^[^ ^73^ ^]^ As shown in the inset of Figure 5f, the ORR current of the IO‐ Ni_x_Co_9‐x_S_8_@NSC electrocatalyst remains stable even after the introduction of 3 m methanol, whereas the current response of Pt/C markedly decreases due to CO poisoning. This observation underscores the superior electrocatalytic durability of the IO‐ Ni_x_Co_9‐x_S_8_@NSC catalyst.

### 2.4 Electrochemical OER Performance of Prepared Electrocatalysts

**Figure 6:**
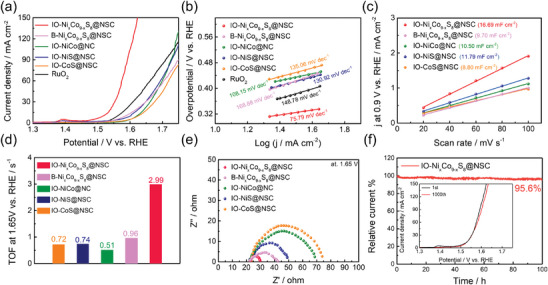
Evaluation of the OER performances of the as‐prepared IO─Ni_x_Co_9‐x_S_8_@NSC, B‐Ni_x_Co_9‐x_S_8_@NSC, IO─NiCo@NC, IO─NiS @NSC, IO‐CoS@NSC, and commercial RuO_2_ a) LSV polarization curves and b) Tafel plots. c) capacitive current densities at different scan rates d) Summarized TOF value at a constant applied potential of 1.65 V versus RHE. e) Nyquist plots obtained from EIS measurements. f) CA response of IO─Ni_x_Co_9‐x_S_8_@NSC at a constant applied potential of 1.52 V versus RHE; the inset shows the polarization curves before and after ADT.

**Figure 7:**
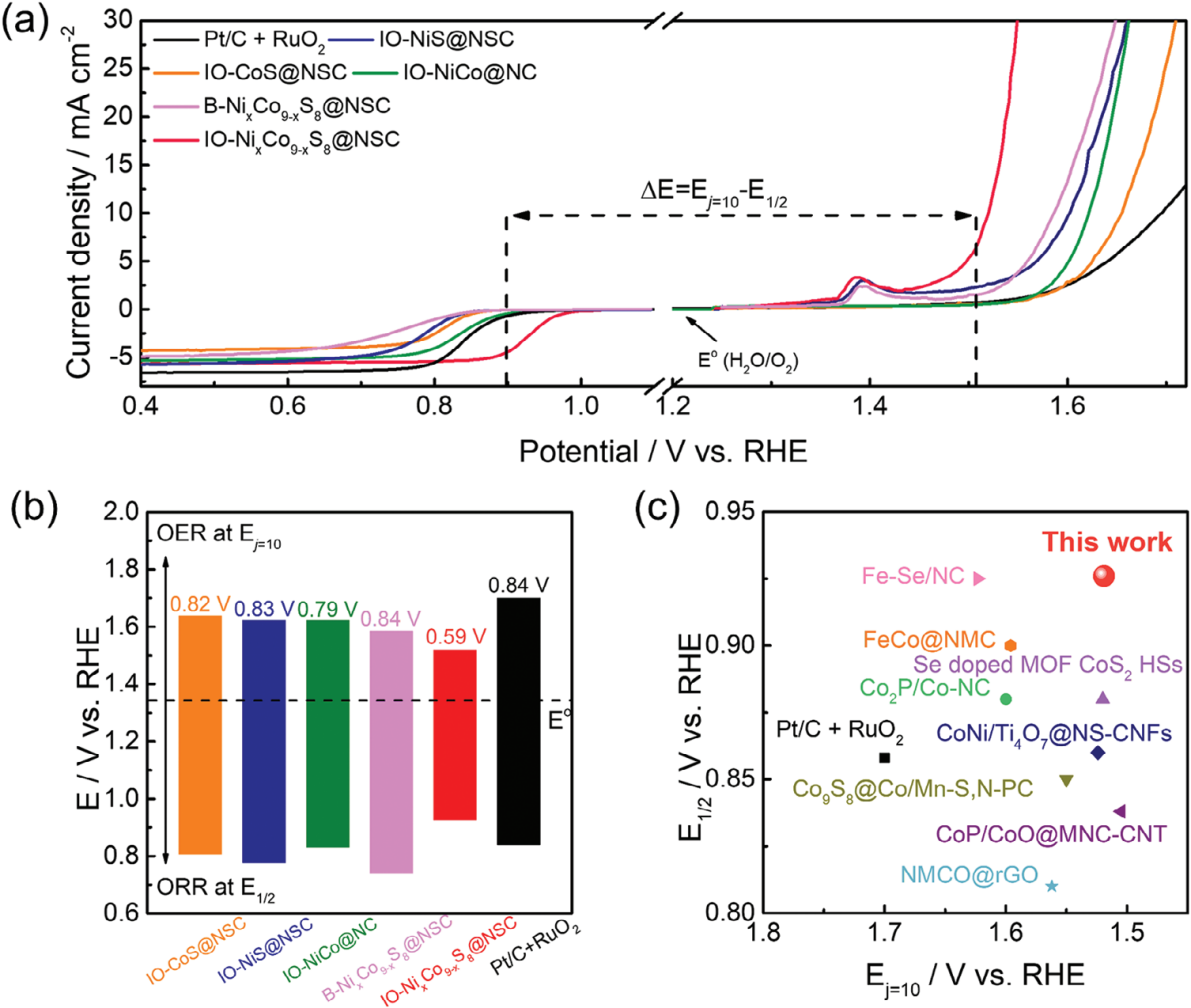
a) LSV polarization curves in the full ORR/OER potential region. b) Calculated potential gap (Δ*E* = *E_j_*_= 10_‐ *E*_1/2_) for IO─Ni_x_Co_9‐x_S_8_@NSC, B‐Ni_x_Co_9‐x_S_8_@NSC, IO─NiCo@NC, IO─NiS@NSC, IO‐CoS@NSC and commercial Pt/C+RuO_2_ catalysts. c) Comparison of bifunctional electrocatalytic activity of IO─Ni_x_Co_9‐x_S_8_@NSC and previously reported noble metal‐free electrocatalysts.

To assess the electrocatalytic activity of the OER, LSV measurements were performed in an N_2_‐saturated 0.1 m KOH electrolyte with a scan rate of 5 mV s^−1^ (**Figure** **6**a). As presented in **Figure** **7**a, the IO─Ni_x_Co_9‐x_S_8_@NSC exhibited the smallest overpotential of 289 mV to achieve a current density of 10 mA cm^−2^ among the B‐Ni_x_Co_9‐x_S_8_@NSC (354 mV), IO─NiCo@NC (392 mV), IO─NiS@NSC (373 mV), IO‐CoS@NSC (397 mV), and precious metal‐based RuO_2_ (332 mV). This enhanced electrocatalytic OER activity is attributed to the synergistic effect between its unique 3D hierarchical structure and the precise defect engineering using abundant heteroatom dopants.^[^ ^22^, ^30^ ^]^ We also examined the OER kinetics of prepared electrocatalysts using the Tafel plot derived from corresponding LSV polarization curves (Figure 6b). The linear Tafel slope of the IO─Ni_x_Co_9‐x_S_8_@NSC sample was calculated to be 75.19 mV dec^−1^, which is the lowest value among the prepared electrocatalysts and commercial RuO_2_. This result demonstrates the outstanding activation characteristics and rapid reaction kinetics of IO─Ni_x_Co_9‐x_S_8_@NSC for alkaline OER. The OER activity of IO─Ni_x_Co_9‐x_S_8_@NSC is found to be significantly superior to those of previously documented transition metal‐based electrocatalysts, as summarized in Table S4 (Supporting Information).

The electrochemically active surface area (ECSA) plays a vital role in determining the effectiveness of electrocatalysis. It is well‐established that the ECSA value and double‐layer capacitance (C_dl_) of an electrocatalyst are directly correlated according to the following equation.

(1) $$ECSA=\frac{C_{\mathrm{dl}}\;\mathrm{of}\;\mathrm{electrocatalyst}\;\left(\mathrm{mF}\;cm^{-2}\right)}{\mathrm{average}\;\mathrm{specific}\;\mathrm{capacitance}\;\left(0.04\;\mathrm{mF}\;cm^{-2}\right)}$$

Consequently, we measured the C_dl_ of prepared electrocatalysts using the cyclic voltammetry (CV) method within the non‐Faradaic potential region of 0.8−1.0 V versus RHE (Figure S13, Supporting Information). All the CV measurements were performed at various scan rates (20−100 mV s^−1^), and capacitive current densities at 0.9 V versus RHE were plotted against the scan rates to calculate the C_dl_ values (Figure 6c). As a result, the IO─Ni_x_Co_9‐x_S_8_@NSC exhibited the highest C_dl_ value of 16.69 mF cm^−2^ among the B‐Ni_x_Co_9‐x_S_8_@NSC (9.70 mF cm^−2^), IO─NiCo@NC (10.50 mF cm^−2^), IO─NiS@NSC (11.79 mF cm^−2^), and IO‐CoS@NSC (8.80 mF cm^−2^). As confirmed by the BET analysis, the geometrical specific surface area of IO─Ni_x_Co_9‐x_S_8_@NSC is substantially smaller than that of IO─NiCo@NC. Nonetheless, the higher *C*_dl_ value of IO─Ni_x_Co_9‐x_S_8_@NSC indicates that the sulfidation process significantly enhanced the electrochemically active surface area. This enhancement can be attributed to the synergistic effects of both the formation of sulfide species and the additional sulfur doping into the carbon matrix. In general, transition metal sulfides exhibit superior charge storage characteristics compared to metallic alloys.^[^ ^74^ ^]^ Moreover, the increased surface polarity resulting from sulfur doping into the carbon matrix facilitates stronger interactions between the electrocatalyst and the electrolyte.^[^ ^36^ ^]^

The intrinsic electrocatalytic activity of the IO─Ni_x_Co_9‐x_S_8_@NSC was further evidenced by turnover frequency (TOF) measurement. In Figure S14 (Supporting Information), the OER LSV polarization curves were converted to TOF curves over the entire potential range. As summarized in Figure 6d, IO─Ni_x_Co_9‐x_S_8_@NSC exhibited a TOF of 2.99 s^−1^ at 1.65 V versus RHE, which is the highest value among B‐Ni_x_Co_9‐x_S_8_@NSC (0.96 s^−1^), IO─NiCo@NC (0.51 s^−1^), IO─NiS@NSC (0.74 s^−1^), and IO‐CoS@NSC (0.72 s^−1^) under identical applied potential. The results suggest that the IO─Ni_x_Co_9‐x_S_8_@NSC catalyst has an extremely low overpotential and improved intrinsic activity per active site for alkaline OER. Moreover, electrochemical impedance spectroscopy (EIS) measurements were conducted to gain deeper insight into the reaction kinetics of the electrocatalysts. The EIS measurement was performed at a constant potential of 1.65 V versus RHE, covering a frequency range from 100 kHz to 100 mHz. The resulting Nyquist plots were fitted using the Randles equivalent circuit model (Figure S15, Supporting Information) and are presented in Figure 6e. Based on the Nyquist plot, the IO─Ni_x_Co_9‐x_S_8_@NSC exhibited a smaller charge transfer resistance (*R*_ct_) of 7.06 Ω than B‐Ni_x_Co_9‐x_S_8_@NSC (19.96 Ω), IO─NiCo@NC (46.16 Ω), IO─NiS@NSC (26.6 Ω), and IO‐CoS@NSC (51.49 Ω), indicating that the introduction of a 3D ordered carbon matrix and heteroatom doping strategy improved the charge transfer efficiency during the OER process. Although B‐Ni_x_Co_9‐x_S_8_@NSC benefits from heteroatom doping, which improves its intrinsic activity, its bulk structure restricts the exposure of active sites. This structural limitation reduces both the electrocatalytic surface area and the mass transfer efficiency, which are crucial for optimal OER performance. As a result, despite having a relatively low R_ct_ value the B‐Ni_x_Co_9‐x_S_8_@NSC exhibited lower OER performance compared to IO‐ Ni_x_Co_9‐x_S_8_@NSC due to the diminished active surface area and mass transfer efficiency. To evaluate the electrochemical durability during the alkaline OER, the CA measurement was conducted using the IO─Ni_x_Co_9‐x_S_8_@NSC at a constant potential of 1.52 V versus RHE (Figure 6f). During continuous operation for 100 h, the relative current response was dropped by only 4.4%, which is a negligible value for large‐scale applications. Moreover, after the consecutive CV scans at a high scan rate of 100 mV s^−1^ (1000 cycles), the OER LSV polarization curve showed only slight degradation, demonstrating outstanding stability for the alkaline OER.

The bifunctional electrocatalytic performance for ORR and OER was assessed by comparing the voltage gaps (Δ*E*) between the half‐wave potential for ORR (*E*_1/2_) and the applied potential at 10 mA cm^−2^ for OER (*E*_j_ = 10). The precious metal‐based electrode was also prepared by mixing the commercial Pt/C and RuO_2_ (Pt:Ru = 50:50 at.%) to serve as a benchmark for bifunctional activity. As shown in Figure 7a,b, the Δ*E* value for IO─Ni_x_Co_9‐x_S_8_@NSC (0.59 V) is notably smaller than B‐Ni_x_Co_9‐x_S_8_@NSC (0.84 V), IO─NiCo@NC (0.79 V), IO─NiS@NSC (0.83 V), IO‐CoS@NSC (0.82 V), and the mixed Pt/C+RuO_2_ catalyst (0.85 V). Additionally, the electrocatalyst prepared in this work showed superior bifunctional activity even when compared to recently reported oxygen‐containing electrocatalysts (Figure 7c). Consequently, it can be concluded that the IO─Ni_x_Co_9‐x_S_8_@NSC electrocatalyst holds significant potential for practical application in rechargeable ZAB.

### 2.5 Application on Rechargeable Zn–Air Battery

**Figure 8:**
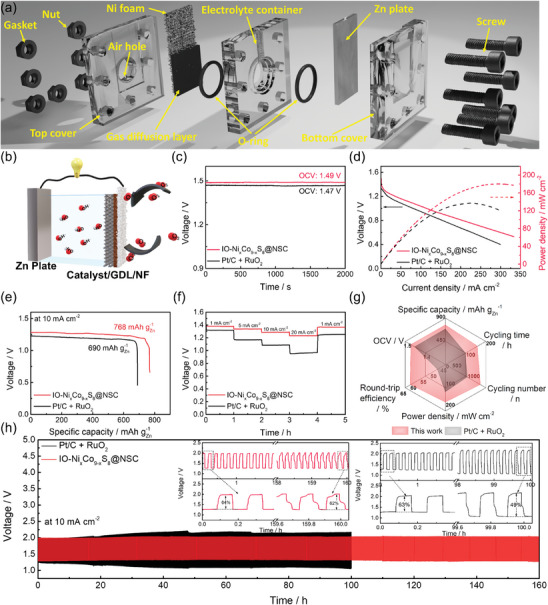
a) Schematic illustration of the ZAB assembly with detailed components. b) Schematic illustration of rechargeable ZAB operation. c) Open circuit voltage, d) discharge polarization and power density curves, and d) galvanostatic full‐discharge curves with a current density of 10 mA cm^−2^. e) Constant current discharge process at different current densities. f) Radar plots with respect to OCV, specific capacity, cycling time, cycling number, power density, and round‐trip efficiency for IO─Ni_x_Co_9‐x_S_8_@NSC and commercial Pt/C+RuO_2_. g) Long‐term galvanostatic charge–discharge process at a pulse current density of 10 mA cm^−2^ with 5 min intervals.

We constructed a homemade ZAB cell to examine the electrochemical performance of the prepared electrocatalyst as the air cathode. **Figure** **8**a illustrates the assembly process of the zinc‐air battery (ZAB) used in this study. As depicted in Figure 8a,b, Ni foam serves as the cathode current collector. The electrocatalyst‐coated gas diffusion layer (GDL) was attached to the Ni foam. As a control sample, the precious metal‐based electrocatalyst was prepared by mixing Pt/C and RuO_2_ with a mass ratio of 1:1. The GDL facilitates efficient oxygen transfer to the active catalytic sites during the ORR. A polished zinc plate, 0.25 mm thick, was employed as the metal anode. The electrolyte container was filled with a 6 m KOH + 0.2 m Zn∙(CH_3_COO)_2_ solution, ensuring a proper supply of reactants to the electrodes. The air electrode was mechanically pressed and dried overnight in a vacuum oven at 50 °C. During ZAB operation, ambient oxygen enters through the air hole and reaches the gas diffusion layer. The top and bottom covers, together with gaskets, O‐rings, and screws, ensure the battery is hermetically sealed. As a result, this custom‐made ZAB cell effectively prevents electrolyte leakage and maintains stable operation throughout the charge–discharge cycles. First, we compared the open circuit voltage (OCV) of ZAB assembled with IO─Ni_x_Co_9‐x_S_8_@NSC and a mixed Pt/C+RuO_2_ electrocatalyst (Figure 8c). The IO─Ni_x_Co_9‐x_S_8_@NSC‐based ZAB exhibited a higher OCV value (1.49 V) than the Pt/C+RuO_2_‐based ZAB (1.47 V). This outcome suggests that the 3D‐ordered structure and high polarity of IO─Ni_x_Co_9‐x_S_8_@NSC not only offer a large active surface area but also facilitate the formation of a three‐phase reaction boundary.^[^ ^34^ ^]^ Figure 8d shows the discharge polarization and corresponding power density curves for the two ZABs. The IO─Ni_x_Co_9‐x_S_8_@NSC‐based ZAB achieved a high peak power density of 180.2 mW cm^−2^, outperforming the Pt/C+RuO_2_‐based ZAB (136.9 mW cm^−2^). Additionally, a galvanostatic full‐discharge test was performed at a constant current density of 10 mA cm^−2^ to evaluate the specific capacities of the ZABs (Figure 8e). The specific capacities, normalized by the weight of the consumed Zn anode, were calculated to be 768 and 690 mAh g_zn_^−1^ for the IO─Ni_x_Co_9‐x_S_8_@NSC and Pt/C+RuO_2_‐based ZABs, respectively. The IO─Ni_x_Co_9‐x_S_8_@NSC‐based ZAB exhibited a 93.7% utilization of theoretical capacity (820 mAh g_zn_^−1^). The outstanding peak power density and specific capacity of the IO─Ni_x_Co_9‐x_S_8_@NSC‐based ZAB are compared with previously reported research and summarized in Table S5 (Supporting Information). When the IO─Ni_x_Co_9‐x_S_8_@NSC‐ZAB was operated with varying current densities rising from 1 to 20 mA cm^−2^, the discharge voltage plateau gradually decreased from 1.38 to 1.23 V, highlighting the outstanding performance of the electrocatalyst under high current density (Figure 8f). In contrast, the Pt/C+RuO_2_‐based ZAB exhibited a serious voltage drop from 1.32 to 0.96 V. After returning the current density to 1 mA cm^−2^, the voltage of IO─Ni_x_Co_9‐x_S_8_@NSC‐based ZAB almost recovered to the initial value (1.36 V), whereas that of the Pt/C+RuO_2_‐based ZAB did not (1.24 V). The galvanostatic charge–discharge cycling test was conducted at 10 mA cm^−2^ with 5 min intervals to assess the electrochemical stability of rechargeable ZAB. As shown in Figure 8h, the IO─Ni_x_Co_9‐x_S_8_@NSC‐based ZAB exhibited steady cycling performance over 160 h (≈960 cycles). We also provided the round‐trip efficiency of the ZABs in the inset of Figure 8h, calculated by dividing the discharge voltage by the charge voltage. Two notable plateaus are observed during the charging process: at ≈1.52 and 1.85 V. These plateaus correspond to the oxidative transitions from Co_9_S_8_ to CoOOH and from CoOOH to CoO_2_, respectively.^[^ ^75^, ^76^ ^]^ Conversely, sequential reduction steps and plateaus are observed during the discharge process, reflecting the transitions from CoO_2_ to CoOOH and subsequently to Co_3_O_4_ (CoO_2_→CoOOH→Co_3_O_4_). At the initial stage of the cycling test, the round‐trip efficiencies of the IO─Ni_x_Co_9‐x_S_8_@NSC and Pt/C+RuO_2_‐based ZABs were calculated to be 64 and 63%, respectively. After 160 h of continuous operation, the round‐trip efficiency of the IO─Ni_x_Co_9‐x_S_8_@NSC‐based ZAB exhibited a slight decrease to 62%. However, the Pt/C+RuO_2_‐based ZAB significantly degraded after only 100 h, exhibiting a round‐trip efficiency of 49%. Even at higher operating current densities (Figure S22, Supporting Information), the IO─Ni_x_Co_9‐x_S_8_@NSC‐based ZAB demonstrated excellent cycling stability, maintaining a high initial round‐trip efficiency of 55%. After 120 h of continuous operation, only a slight efficiency degradation of ≈5% was observed, confirming the physicochemical durability of the prepared electrocatalyst. In contrast, the Pt/C+RuO_2_‐based ZAB exhibited significantly lower round‐trip efficiencies, starting at 36% and dropping to 27% after only 33 h of operation. These cycling performance tests highlight the superior reversibility and electrochemical stability of the IO─Ni_x_Co_9‐x_S_8_@NSC electrocatalyst during the charge–discharge process in harsh alkaline conditions. The cycling performance test reveals the superior reversibility and electrochemical stability of the prepared electrocatalyst during the charge–discharge process of the ZAB in harsh alkaline conditions.

In summary, the physicochemical and electrochemical analyses presented above demonstrate that the prepared IO─Ni_x_Co_9‐x_S_8_@NSC exhibits exceptional electrocatalytic activity and durability, making it a highly promising catalyst for both ORR and OER. This achievement is attributed to the synergistic effects of several key features. First, the unique 3D inverse‐opal structure offers a large electrochemically active surface area and mechanically stable architecture, enhancing mass transfer efficiency and the accessibility of active sites during electrocatalysis.^[^ ^31^, ^32^ ^]^ Second, the self‐sacrificial silica template with surface treatment promotes the homogeneous dispersion of Ni_x_Co_9‐x_S_8_ nanocrystals, preventing aggregation and increasing the number of exposed active sites for both ORR and OER.^[^ ^28^, ^29^ ^]^ Additionally, the introduction of Ni dopants into the Co_9_S_8_ structure induces significant lattice distortion and defect sites, which serve as highly efficient active sites for alkaline ORR and OER.^[^ ^25^ ^]^ Finally, the uniform distribution of nitrogen and sulfur heteroatoms effectively disrupts the electro‐neutrality of the sp^2^ carbon structure and alters the electron spin density, thereby enhancing the surface polarity of the electrocatalyst. This improves the interaction between reactants and active sites on the catalyst layer during electrocatalysis.^[^ ^77^, ^78^ ^]^ As a result, the combined influence of these factors leads to the remarkable performance of IO─Ni_x_Co_9‐x_S_8_@NSC in ORR, OER, and even in rechargeable ZAB applications.

## 3 Conclusions

Nickel‐doped Co_9_S_8_ nanoparticles dispersed on N, S co‐doped porous carbon have been successfully prepared by self‐assembly, annealing, template etching, and sulfidation process. NixCo_9‐x_S_8_@NSC displayed remarkable activity with a lower overpotential and Tafel slope compared to other synthesized samples and commercial RuO_2_. The catalyst showed high electrochemically active surface area (ECSA) and turnover frequency (TOF), indicating enhanced intrinsic activity. The bifunctional electrocatalytic performance of IO─Ni_x_Co_9‐x_S_8_@NSC was highlighted by its small voltage gap between ORR and OER, suggesting its potential for practical application in ZABs. Indeed, ZABs assembled with IO─Ni_x_Co_9‐x_S_8_@NSC as the air cathode exhibited higher open circuit voltage (OCV), peak power density, and specific capacity compared to those with a commercial Pt/C+RuO_2_ catalyst. The IO─NixCo_9‐x_S_8_@NSC‐based ZAB demonstrated excellent performance and stability even under high current densities and prolonged cycling tests. Overall, the synthesized IO─Ni_x_Co_9‐x_S_8_@NSC electrocatalyst holds great promise for efficient and durable electrochemical energy conversion and storage devices, particularly in rechargeable ZABs, owing to its outstanding catalytic activity, stability, and methanol tolerance. These findings contribute to the advancement of electrocatalysis for clean and sustainable energy technologies.

## Conflict of Interest

The authors declare that they have no conflict of interest.

## Supporting information



Supporting Information

## Data Availability

The data that support the findings of this study are available from the corresponding author upon reasonable request.
