# Supplementary material for: Innovative Air Cathode with Ni‐Doped Cobalt Sulfide in Highly Ordered Macroporous Carbon Matrix for Rechargeable Zn–Air Battery
Source: Adv Sci (Weinh). 2024 Oct 14;11(45):2407915. doi: 10.1002/advs.202407915 (PMC11615759; doi:10.1002/advs.202407915)
Supplement: Supplementary file 1 — Supporting Information [file ADVS-11-2407915-s001.pdf]

ADVANCED SCIENCE
Open Access

Supporting Information

for *Adv. Sci.*, DOI 10.1002/advs.202407915

Innovative Air Cathode with Ni-Doped Cobalt Sulfide in Highly Ordered Macroporous Carbon Matrix for Rechargeable Zn–Air Battery

*Yujin Son, Kyeongseok Min, Sungkyun Cheong, Boyoung Lee, Sang Eun Shim and Sung-Hyeon Baeck**

Innovative Air Cathode with Ni-Doped Cobalt Sulfide in Highly Ordered Macroporous Carbon Matrix for Rechargeable Zn-Air Battery

*Yujin Son[1], Kyeongseok Min[1], Sungkyun Cheong, Boyoung Lee, Sang Eun Shim, and Sung-Hyeon Baeck**

Department of Chemistry and Chemical Engineering, Education and Research Center for Smart Energy Materials and Process, Inha University, Incheon 22212, Republic of Korea

**Corresponding author*

E-mail address: shbaeck@inha.ac.kr

[1]*These authors contributed equally to this work.*

Experimental section

1. Materials and chemicals

Tetraethyl orthosilicate (TEOS, $C_8H_{20}O_4Si$, 98%), 3-Aminopropyltrimethoxysilane (APTMS, $H_2N(CH_2)_3Si(OCH_3)_3$ 97%), Cobalt (II) nitrate hexahydrate ($Co(NO_3)_2{\cdot}6H_2O$, 98%), Nickel (II) nitrate hexahydrate ($Ni(NO_3)_2{\cdot}6H_2O$, 99%), thiourea (CH_4N_2S, 99%), Polyvinylpyrrolidone ($(C_6H_9NO)_x$, K30), Nafion perfluorinated resin solution (5 wt.%) were procured from Sigma-Aldrich (USA). Sodium hydroxide (NaOH, 98%) was sourced from Samchun Co. (Korea), while ammonia water (NH_4OH, 25-29%) was acquired from DUKSAN Co. (Korea). 20 wt.% Pt/C was purchased from Alfa Aesar (USA). All materials were utilized as received without additional purification.

2. Synthesis of NH_2-SiO_2

SiO_2 nanospheres were synthesized through a modified Stober method. Initially, a mixture comprising 12.8 mL of tetraethyl orthosilicate (TEOS), 200 mL of ethanol (EtOH), 18.4 mL of deionized water (DI water), and 13 mL of ammonium hydroxide (NH_4OH) was vigorously stirred using a magnetic stirrer for 12 h at ambient temperature. The resultant white solution underwent successive washing steps with ethanol and deionized water to eliminate residual impurities. Subsequently, 1 g of SiO_2 was dispersed ultrasonically in a solution consisting of 180 mL ethanol and 1.5 mL APTMS, followed by stirring at 80 °C for 12 h. The resulting product was then collected through centrifugation. The resulting product was denoted as 'NH_2-SiO_2'.

3. Synthesis of IO-$Ni_xCo_{9-x}S_8$@NSC

The prepared NH_2-SiO_2 (0.5g) was dispersed ultrasonically in 20 mL of DI water. Subsequently, 66 mg of $Ni(NO_3)_2{\cdot}6H_2O$, 132 mg of $Co(NO_3)_2{\cdot}6H_2O$ (with molar Ni:Co ratio of 1:2), and

400mg of PVP were added to the solution. Following this, the obtained pink solution underwent washing via vacuum filtration to obtain a closely packed opal template, designated as 'NiCo-NH_2-SiO_2/PVP'. The NiCo-NH_2-SiO_2/PVP composite was annealed in a tube furnace at 650°C with a heating rate of 5°C min^{-1} for 3h under an Ar atmosphere, yielding the product denoted as 'NiCo-NH_2-SiO_2@NC'. To achieve the inverse opal structure, the silica spheres within NiCo-NH_2-SiO_2@NC were leached in a 2 M NaOH solution resulting in 'IO-NiCo@NC'. Finally, 40 mg of IO-NiCo@NC and 1.2 g of thiourea were positioned downstream and upstream of the tube furnace, respectively. The tube furnace was then heated to 550°C with a heating rate of 5°C min^{-1} for 3h under an Ar atmosphere. The resultant product was designated as 'IO-$Ni_xCo_{9-x}S_8$@NSC'. Similarly, bulk-structured $Ni_xCo_{9-x}S_8$@NSC (B-$Ni_xCo_{9-x}S_8$@NSC) was synthesized following the same procedure but without the utilization of SiO_2 sphere. Additionally, monometallic IO-NiS@NSC and IO-CoS@NSC samples were prepared using the identical synthetic procedure as that for IO-$Ni_xCo_{9-x}S_8$@NSC. The quantity of moles of metal species in both IO-NiS@NSC and IO-CoS@NSC samples matched that of IO-$Ni_xCo_{9-x}S_8$@NSC.

4. Physicochemical characterization

X-ray diffraction (XRD) spectrometer (Rigaku, D/Max 220 V/PC; Inha University Core-Facility Center for Sustainable Energy Materials (CFSE) of Korea Basic Science Institute (KBSI)) using Cu-Kα radiation (λ = 0.154056 nm) was utilized to validate the phase composition and crystal structure of the synthesized materials. Fourier-transform infrared (FT-IR) spectroscopy (Bruker VERTEX 80V spectrometer) was conducted to evaluate the chemical bonding and functional groups of prepared electrocatalysts. The zeta-potential of prepared sample was measured using zeta potential analyzer (Malvern, Nano Zetasizer). Raman spectra of the prepared samples were obtained using a HORIBA LabRAM HR Evolution instrument

with an excitation light source (λ = 532 nm). The Raman spectrum was obtained via a HORIBA LabRAM HR Evolution instrument with an excitation wavelength of 532 nm, a power of 10 mW. Various advanced microscopy and analytical tools were employed to study the sample morphologies and internal structures. All the morphological details were captured on a S-4300SE field-emission scanning electron microscope (FESEM) operating at 15 kV and a HEM2100F transmission electron microscope (TEM) with an operational voltage of 200 kV. The elemental content was investigated using inductively coupled plasma optical emission spectrometry (ICP-OES, Optima 7300DV) for the determination of Ni and Co. Elemental analysis of N, S, and C was performed using an EA1112 CHNOS elemental analyzer. Brunauer–Emmett–Teller (BET) and Barrett–Joyner–Halenda (BJH) analysis methods were used to calculate the specific surface area and pore size distribution of the samples, respectively, using nitrogen adsorption–desorption experiments at 77 K (ASAP 2020). The contact angle was measured by Data-Physics Instrument (DCAT 21). Finally, the element and electronic state of the sample was performed via X-ray photoelectron spectroscopy (XPS, K-alpha+, ThermoFisher Scientific) with monochromatic Al-Kα radiation (hν = 1486.6 eV).

5. Electrochemical half-cell test for ORR and OER

All of the electrochemical measurements were conducted using an electrochemical workstation (Princeton Applied Research, VSP potentiostat). A typical three-electrode system was used, comprising a graphite counter electrode, glassy carbon (GC) working electrode, and Hg/HgO reference electrode at room temperature. The electrocatalyst ink was prepared by adding 2 mg of the catalyst, 16 μL of a 5 wt% Nafion solution, and 100 μL of isopropyl alcohol to 384 μL of DI water. After ultrasonic treatment of the solution for 30 min, 5 μL of the obtained catalyst ink was loaded onto the GC electrode (geometric area = 0.0707 cm^2). Therefore, the loading mass of prepared electrocatalyst was designed to be about 0.3 mg cm^{-2}. Linear

scanning voltammetry (LSV) curves were obtained at a constant scan rate of 5 mV s^{-1} in O_2- and N_2-saturated 0.1 M KOH electrolyte for the ORR and OER, respectively. During the measurements, the LSV curves were corrected using 85% *iR*-compensation. All of the measured potentials were converted to RHE potentials according to the following Nernst equation:

$$E_{RHE} = E_{Hg/HgO} + 0.0592 \times pH + E^0_{Hg/HgO}.$$

The $E^0_{Hg/HgO}$ value was 0.098 V vs. RHE, which was measured via calibration using a stable SCE electrode. The Koutecky–Levich (K-L) equation was used to calculate the ORR parameters. The number of electrons transferred during the ORR process was estimated from the LSV polarization curves measured at various rotation speeds using the K-L equation, as follows:

$$\frac{1}{J} = \frac{1}{J_K} + \frac{1}{J_D},$$

$$J_D = 0.62nFD^{2/3}\nu^{-1/6}\omega^{1/2}C_{O_2},$$

where J is the measured current density; J_K and J_D are the kinetic- and diffusion-limiting current densities, respectively; n is the number of electrons transferred; F is the Faraday constant (96485 C mol^{-1}); D is the diffusion coefficient of O_2 in 0.1 M KOH (1.93×10^{-5} cm^2 s^{-1}); ν is the kinematic viscosity (1.09×10^{-2} cm^2 s^{-1}); ω is the angular frequency of the RDE ($\omega = 2\pi f$ / 60, where f is the rotating speed of the RDE in rpm); and C_{O_2} is the concentration of O_2 in 0.1 M KOH (1.2×10^{-6} mol cm^{-3}). Rotating ring-disc electrode (RRDE) measurements were performed at a scan rate of 5 mV s^{-1} using the same catalyst inks used for the RDE measurements. The number of electrons transferred (n) and peroxide yield (%HO_2^-) were estimated using the following equations:

$$n = 4 \times \frac{I_d}{I_d + I_r \times N},$$

$$\%HO_2^- = 200 \times \frac{I_d \times N}{I_d + I_r \times N},$$

where I_d and I_r are the disc and ring currents, respectively, and N represents the collection efficiency of the Pt ring (0.37). Electrochemical impedance spectroscopy (EIS) measurements were recorded in the frequency range of 100 kHz to 100 mHz to calculate the charge transfer resistance (R_{ct}) of the prepared electrocatalysts. The turnover frequency (TOF) was calculated using the following equation:

$$\mathrm{TOF} = \frac{jA}{zFn},$$

where j is the current density (mA cm^{-2}) during the LSV measurement, A represents the geometric area of the GC (0.0707 cm^2), z is the number of electrons transferred (four for the OER), F is the Faradaic constant (96485 C mol^{-1}), and n is the number of active sites. The n values of as-prepared samples were determined from CV tests in the potential range of −0.2 to 0.6 V vs. RHE in 1.0 M phosphate buffered saline (PBS) solution according to the following equation:

$$n = \frac{Q}{2F},$$

where Q is the total charge accompanying the CV measurement. To calculate the double-layer capacitance (C_{dl}), CV measurements were conducted in the non-Faradaic potential region from 0.8 to 1.0 V vs. RHE at different scan rates (20−100 mV s^{-1}). The C_{dl} value was estimated by calculating the slope of the linear fitting plot of the capacitive current densities in the CV tests against the scan rate. The electrochemical active surface area (ECSA) values of the electrocatalysts were calculated by dividing C_{dl} by the specific capacitance (C_S, 40 µF in alkaline solution). The long-term electrocatalytic durability of electrocatalyst was estimated using chronoamperometry (CA) at a constant potential of 0.7 and 1.59 V vs. RHE for the ORR and OER, respectively.

6. Fabrication and performance test of rechargeable Zn–air battery

A home-made rechargeable ZAB was assembled using IO-$Ni_xCo_{9-x}S_8$@NSC or Pt/C + RuO_2 (mass ratio of 1:1) electrocatalysts as the air cathode. To prepare the air electrode, a catalyst slurry was prepared by mixing the catalyst samples (10 mg), 5 wt% Nafion solution (5 μL), and DI water (500 μL). The prepared slurry was then slowly dropped onto carbon paper (loading density: 1 mg cm^{-2}) and dried in a vacuum oven at 50 °C for 30 min. The air electrode was mechanically pressed and dried overnight in a vacuum oven at 50 °C. Additionally, 0.2 M Zn·$(CH_3COO)_2$ was completely dissolved in a 6.0 M KOH solution and employed as the electrolyte for the ZAB. A polished zinc plate with a thickness of 0.25 mm was used as the metal anode. The charge/discharge polarization curves of the as-assembled ZABs were obtained by varying the voltage within the selected cut-off region at a constant scan rate of 10 mV s^{-1}. Galvanostatic charge/discharge measurements were conducted using a battery test station (WonATech, WMPG1000) at room temperature and ambient air conditions. A long-term cyclability test was performed with a pulse current density of 10 mA cm^{-2} for 5 min during each charge/discharge step.

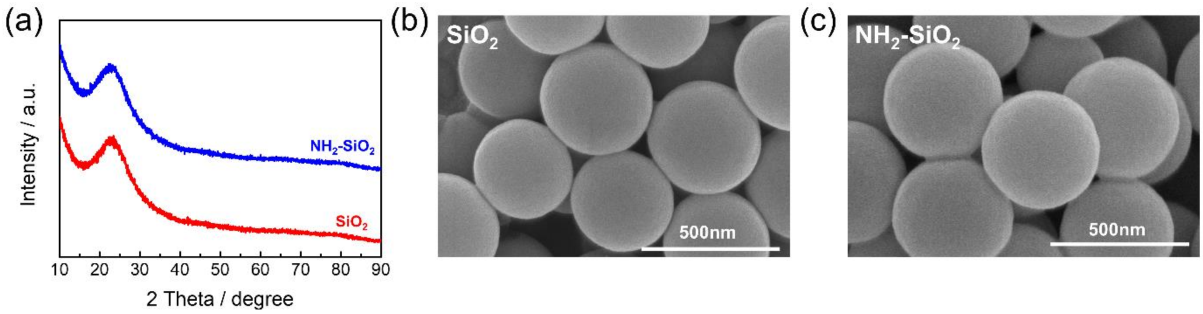

Figure S1. (a) X-ray diffraction (XRD) patterns of SiO_2. Scanning electron microscopy (SEM) images of (b) SiO_2 and (c) NH_2-SiO_2.

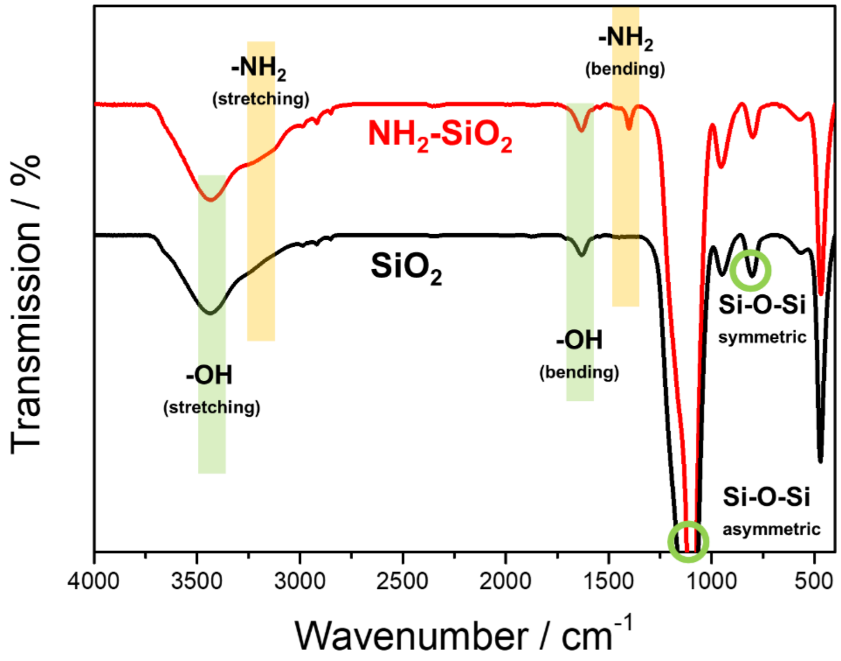

Figure S2. Fourier-transform infrared (FT-IR) spectra of SiO_2 and NH_2-SiO_2 within 4000−400 cm^{-1}.

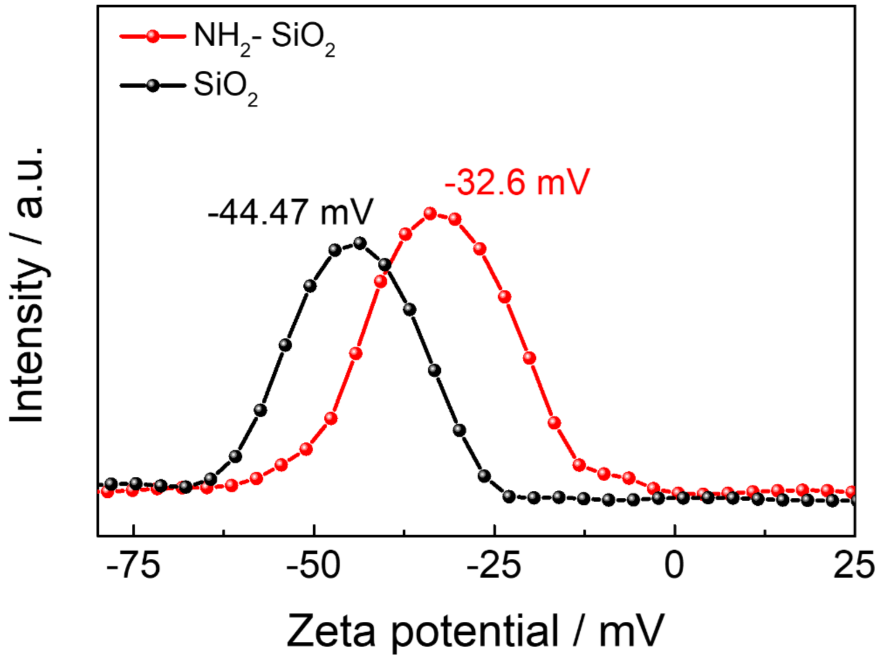

Figure S3. Zeta potential distribution of NH_2-SiO_2 and SiO_2 dispersed in DI water.

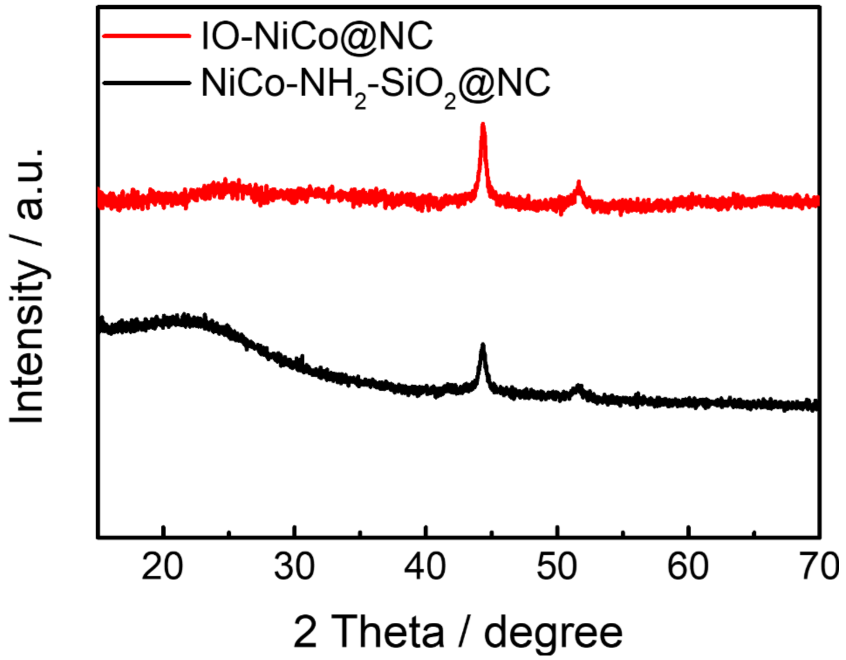

Figure S4. X-ray diffraction (XRD) patterns of before(black line) and after(red line) removal of silica.

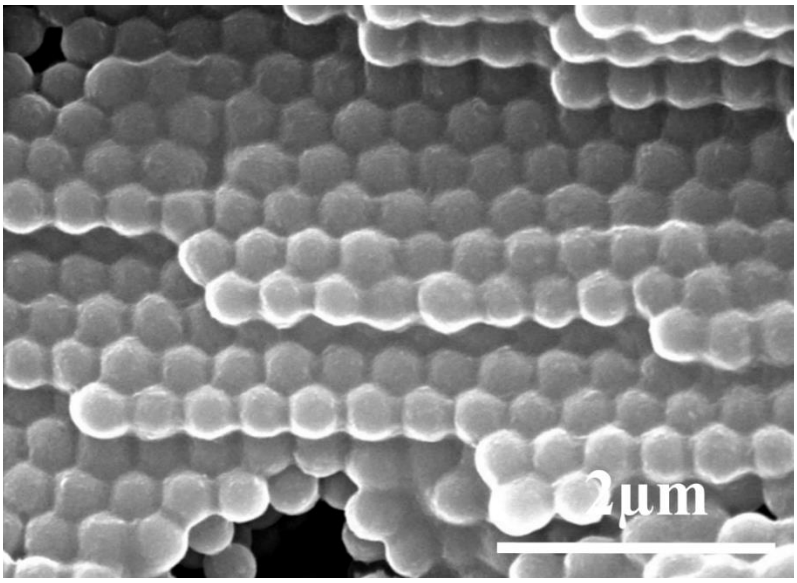

Figure S5. Scanning electron microscopy (SEM) images of uniform opal template (NiCo-NH_2-SiO_2/PVP).

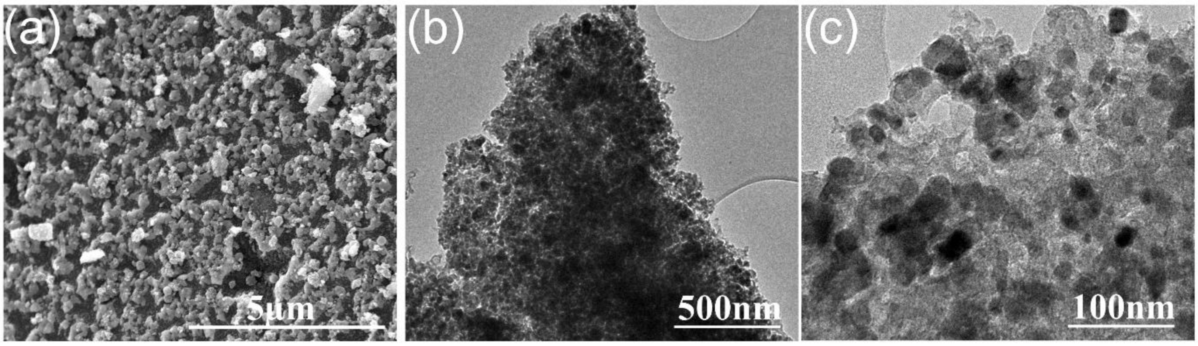

Figure. S6. (a) Scanning electron microscopy (SEM). (b) and (c) Transmission electron microscopy (TEM) images of B-$Ni_xCo_{9-x}S_8$@NSC.

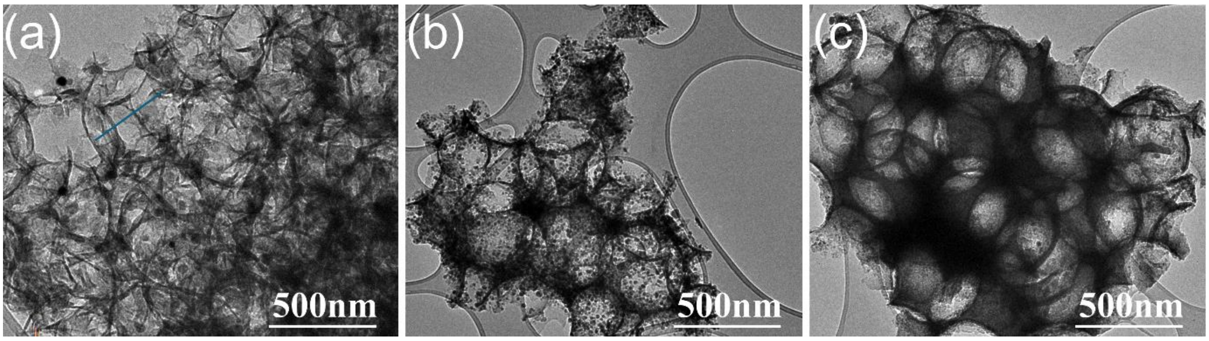

Figure S7. Transmission electron microscopy (TEM) images of (a) IO-NiCo@NC (b) IO-NiS@NSC, (c) IO-CoS@NSC.

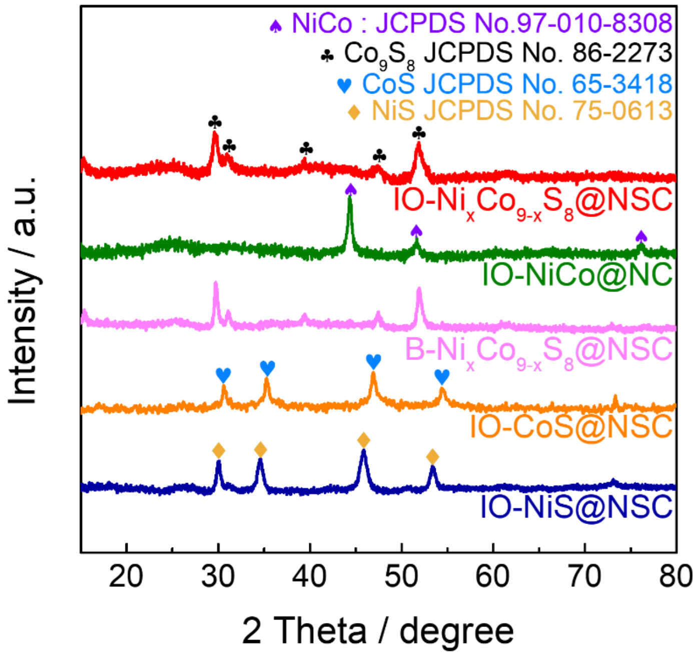

Figure S8. X-ray diffraction (XRD) patterns of all the prepared samples.

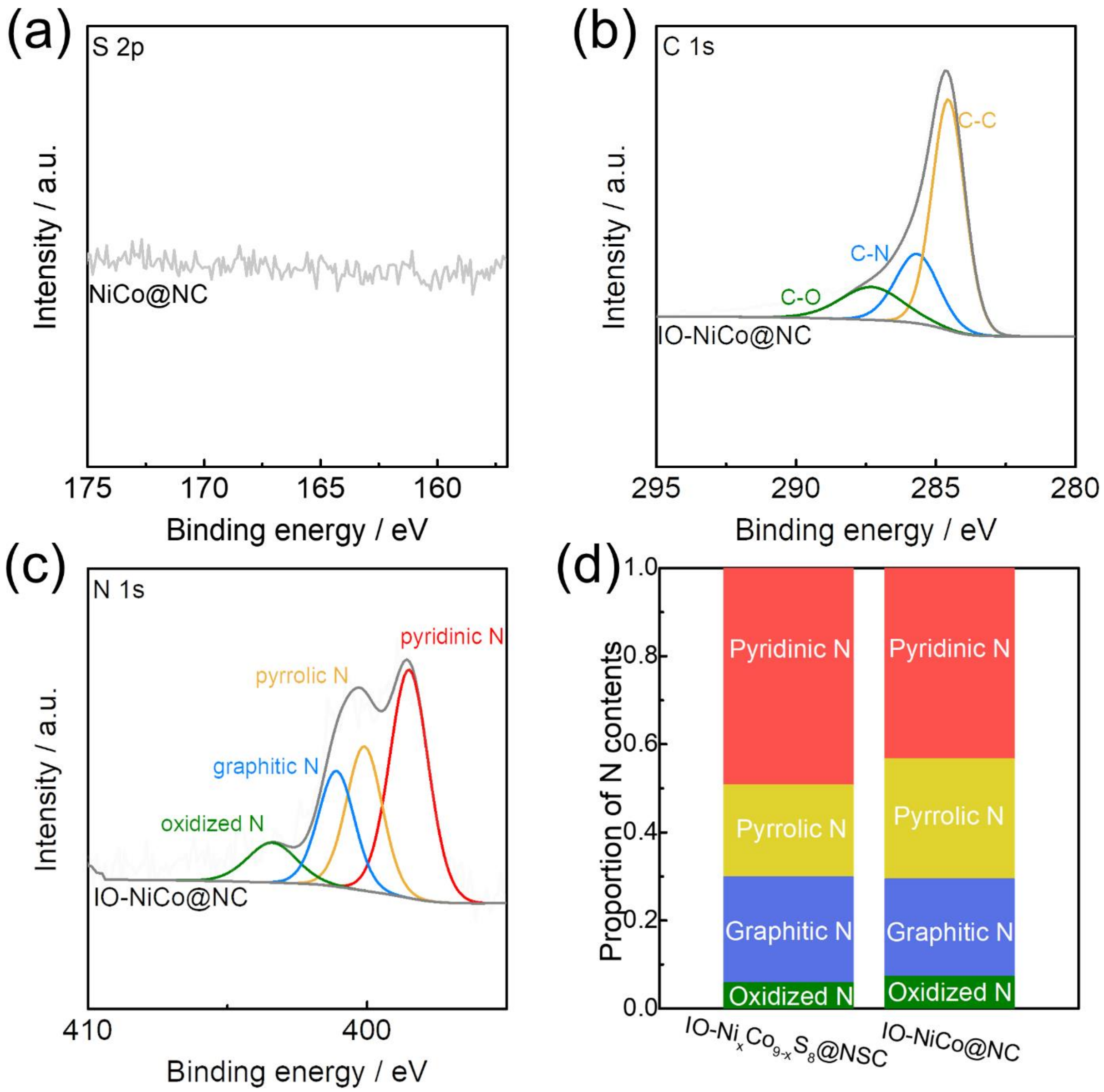

Figure S9. High-resolution XPS spectra of (a) S 2p, (b) C 2p, and (c) N 1s for IO-$Ni_xCo_{9-x}S_8$@NSC and IO-NiCo@NC samples. (d) Relative proportions of different N species in IO-$Ni_xCo_{9-x}S_8$@NSC and IO-NiCo@NC.

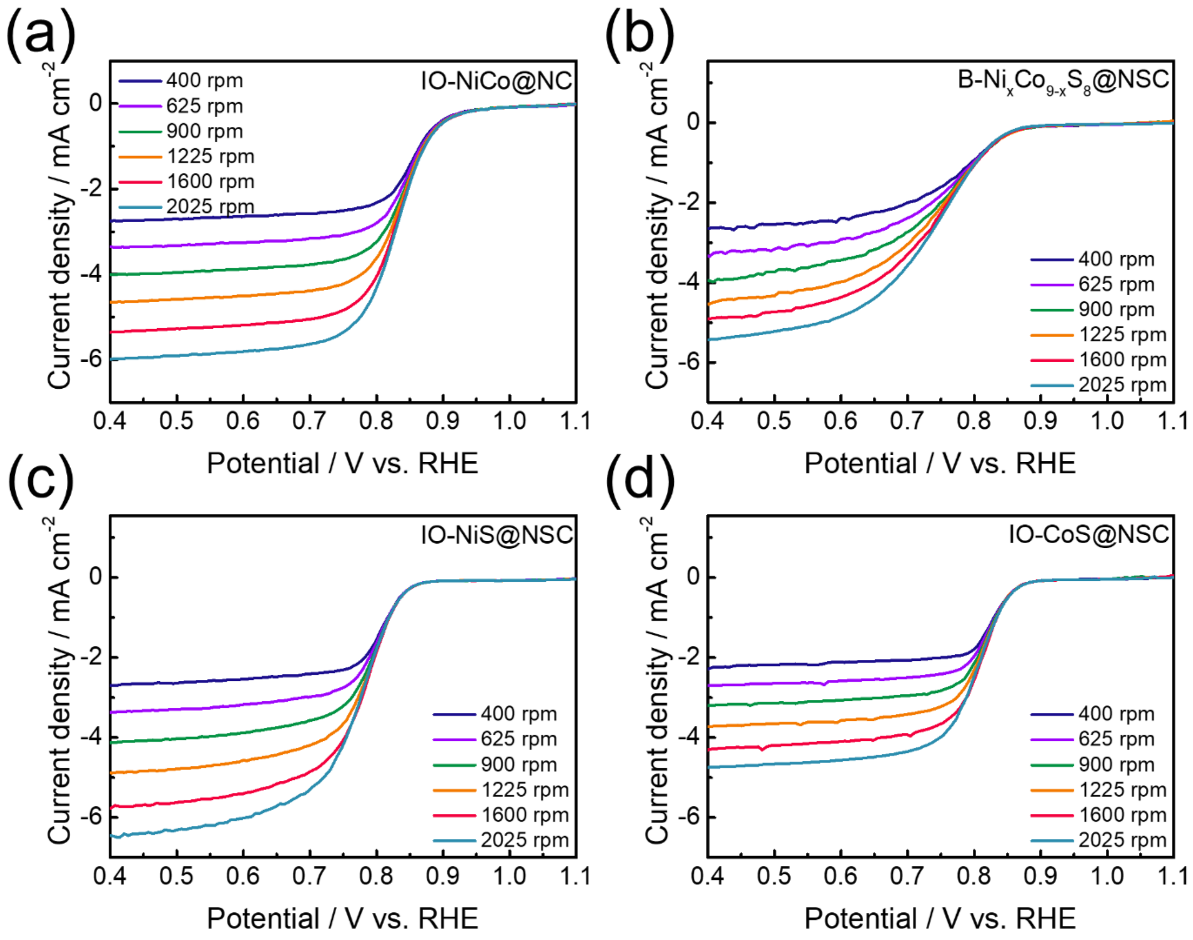

Figure S10. Rotating disk voltammograms of (a) IO-NiCo@NC, (b) B-$Ni_xCo_{9-x}S_8$@NSC, (c) IO-NiS@NSC, and (d) IO-CoS@NSC samples.

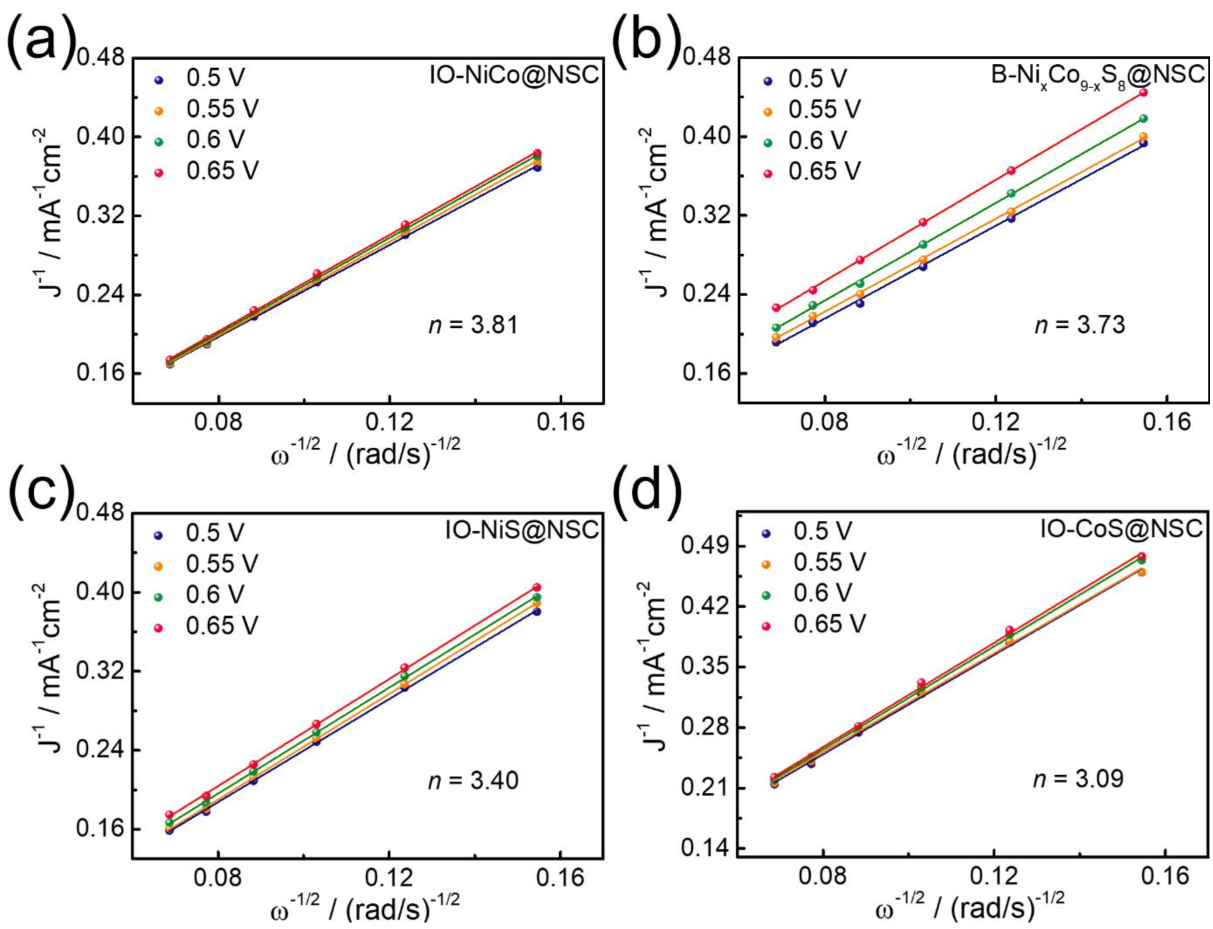

Figure S11. Koutecky-Levich plots of (a) IO-NiCo@NC, (b) B-$Ni_xCo_{9-x}S_8$@NSC, (c) IO-NiS@NSC, and (d) IO-CoS@NSC samples obtained from Figure S10.

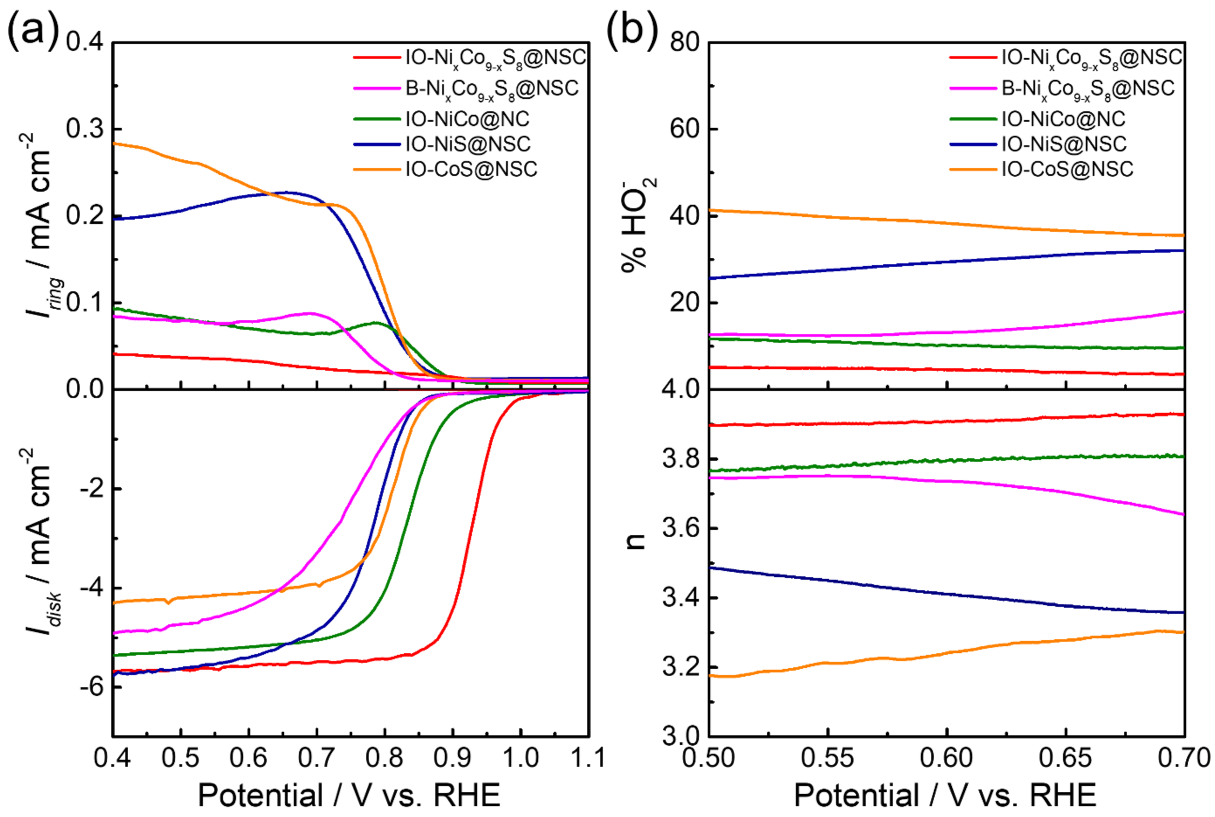

Figure S12. (a) Ring and disk current obtained from RRDE measurement and (b) corresponding HO_2^- yield and *n* values for as-prepared electrocatalysts.

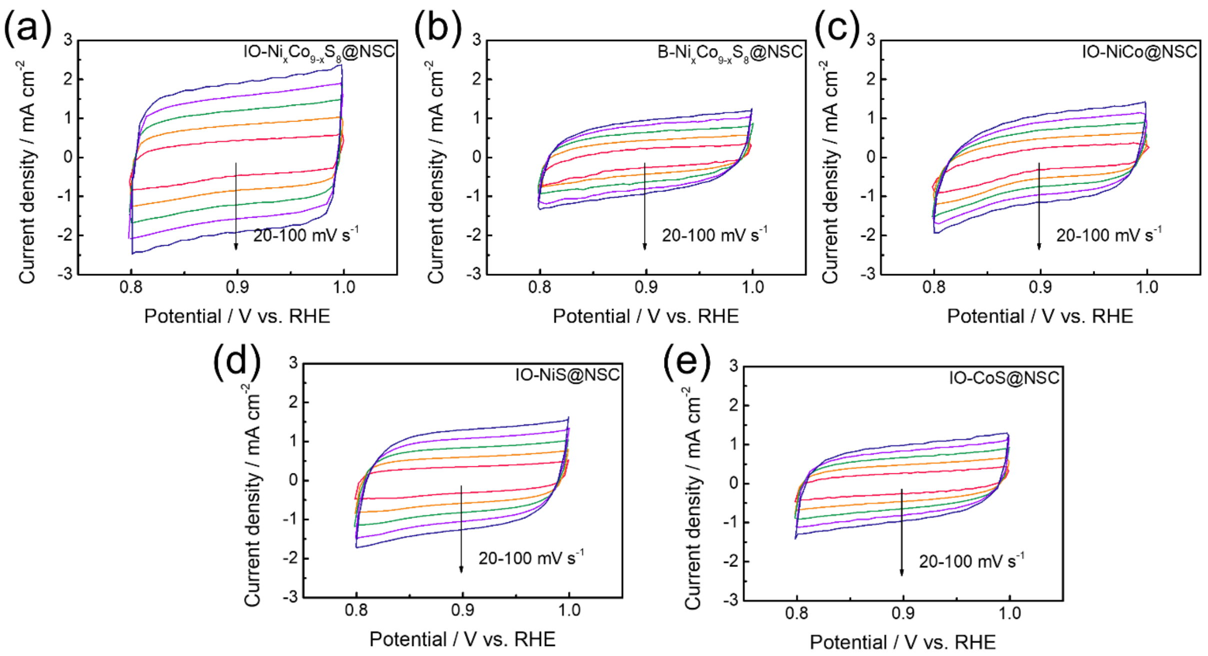

Figure S13. CV curves of (a) IO-$Ni_xCo_{9-x}S_8$@NSC, (b) B-$Ni_xCo_{9-x}S_8$@NSC, (c) IO-NiCo@NC, (d) IO-NiS@NSC, and (e) IO-CoS@NSC measured in non-faradaic potential region.

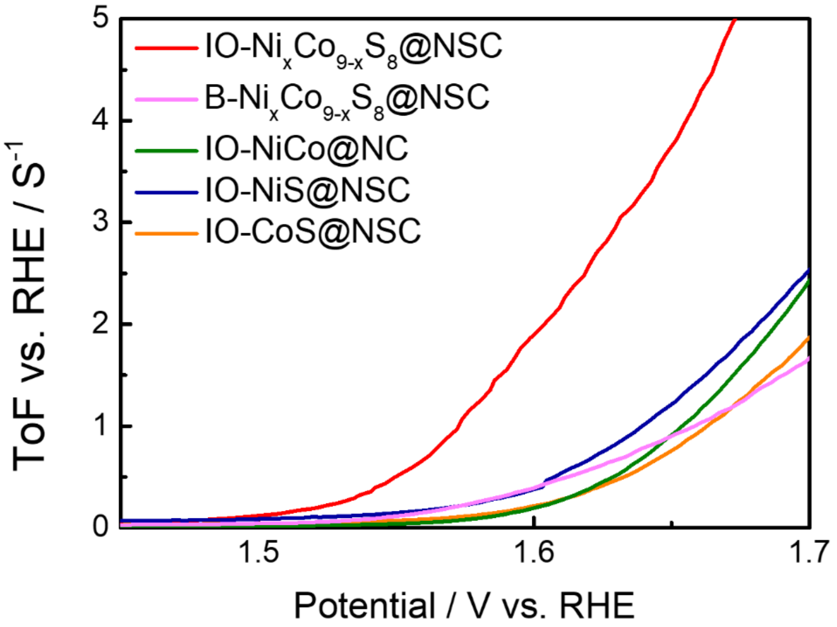

Figure S14. Electrocatalytic measurement of prepared electrocatalysts for the TOF curves obtained by conversion of corresponding polarization curves.

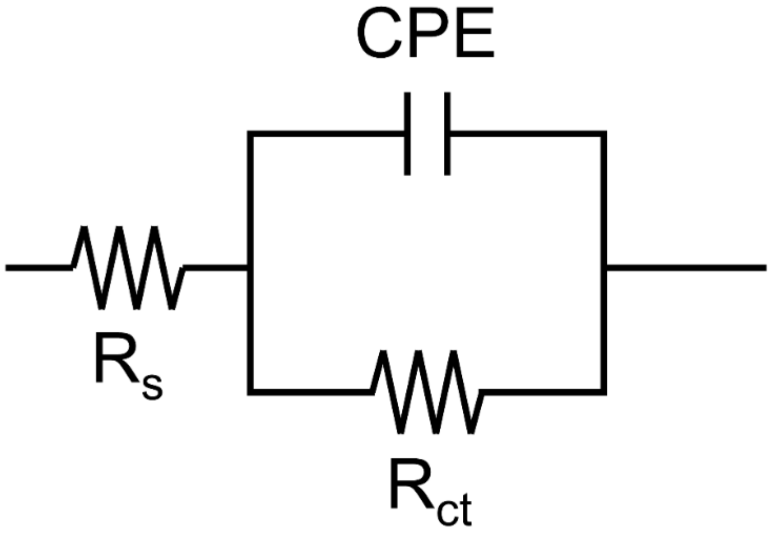

Figure S15. Equivalent circuit model (Randles circuit) consist of solution resistance (R_s), constant phase element (CPE), and charge-transfer resistance (R_{ct}) for fitting of EIS plot.

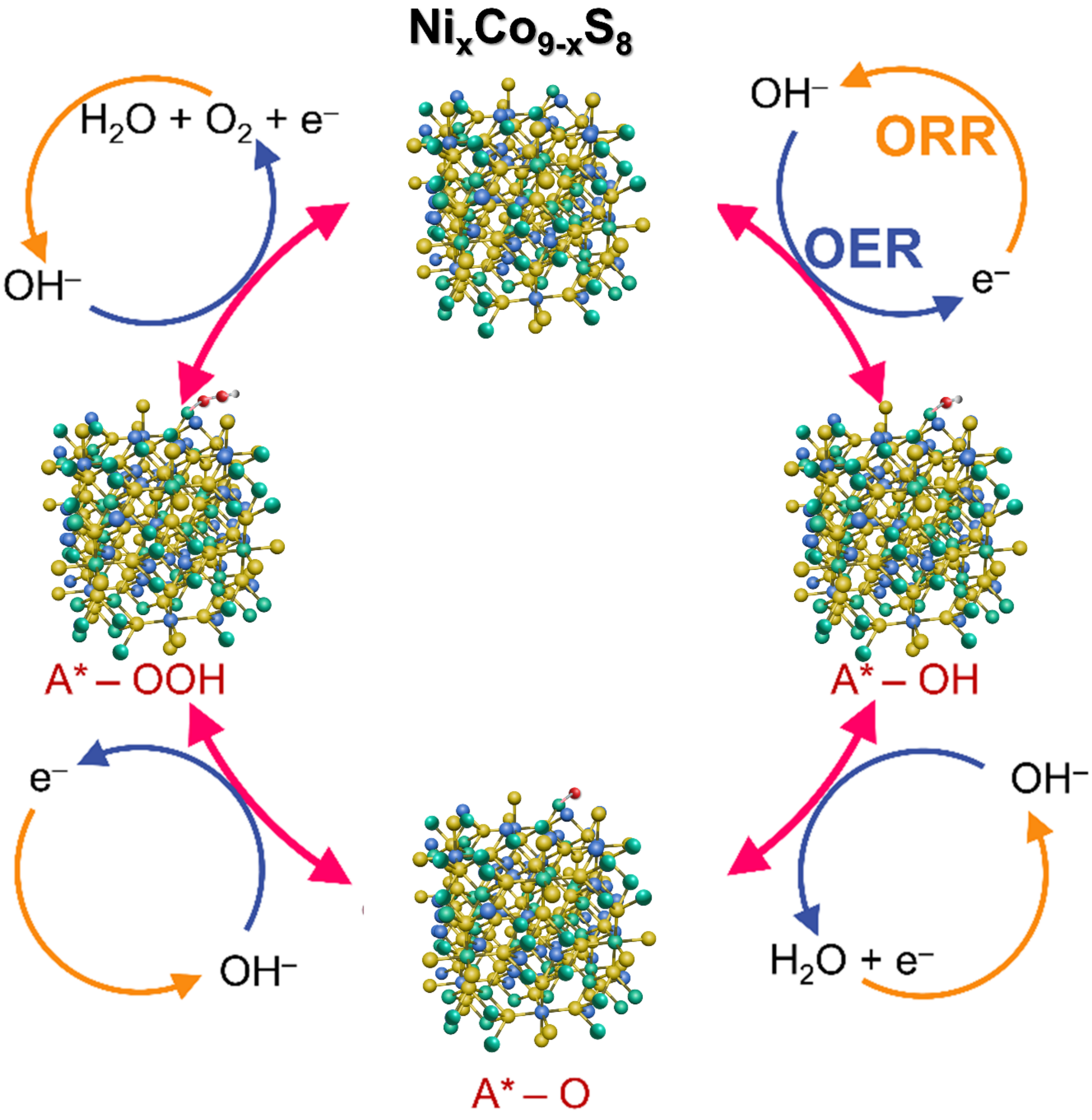

Figure S16. Schematic illustration of the ORR and OER pathway of the $Ni_xCo_{9-x}S_8$ (A* = active sites).

Physicochemical analysis after the stability test for ORR and OER

Following the long-term stability tests for ORR and OER using the IO-$Ni_xCo_{9-x}S_8$@NSC electrocatalyst, surface morphology and chemical structure were examined through SEM and Raman analyses. As shown in Figure S17a and b, the post-ORR SEM image reveals that the IO-$Ni_xCo_{9-x}S_8$@NSC retains its inverse opal structure under alkaline ORR conditions, with only minimal surface roughening observed. In contrast, the post-OER sample displayed more pronounced structural collapse and surface roughening compared to the post-ORR sample, likely due to the vigorous gas evolution during the OER process. Nonetheless, the porous framework remained largely intact after the 100 h OER test, demonstrating the structural durability of the electrocatalyst.

Raman analysis of the post-ORR sample (Figure S17c) revealed no significant signals within the Raman shift range from 400 to 1200 cm^{-1}, further indicating the physicochemical stability of the electrocatalyst under alkaline ORR conditions. In comparison, the Raman spectrum of the post-OER sample exhibited distinct peaks at 554 and 650 cm^{-1}, corresponding to the formation of CoOOH species. Additionally, peaks at 472 and 1150 cm^{-1} were attributed to NiOOH and O−O bonding, respectively, confirming the surface reconstruction of the electrocatalyst into metal oxyhydroxides during the alkaline OER process.[1-4] These *in-situ* formed metal oxyhydroxide species are generally recognized as highly active sites for OER in alkaline media, which contributes to the enhanced electrocatalytic performance.[5]

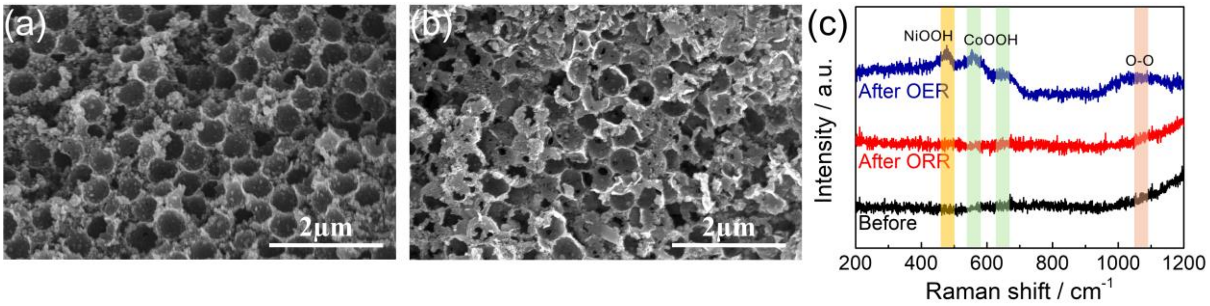

Figure S17. Scanning electron microscopy (SEM) of IO-$Ni_xCo_{9-x}S_8$@NSC electrocatalyst after (a) ORR and (b) OER durability test. (c) Raman spectra of IO-$Ni_xCo_{9-x}S_8$@NSC after the stability test.

IO-$Ni_xCo_{9-x}S_8$@NSC prepared with various synthetic conditions

To investigate the effect of sulfidation temperature on the catalyst, we annealed the IO-NiCo@NC with the thiourea at various temperature of 400, 450, 500, 550, and 600°C. First, the X-ray diffraction (XRD) measurement was conducted to explore the change in crystal structure with respect to the different sulfidation temperature (Figure S18). The characteristic peaks for the NiCo alloy were completely disappeared by thermal sulfidation. At the low sulfidation temperature, the metal sulfide exists as $NiCo_2S_4$ spinel structure. Along with the increase of sulfidation temperature, the crystal structure of metal sulfide changes from $NiCo_2S_4$ to $Ni_xCo_{9-x}S_8$ through a metastable $Ni_xCo_{1-x}S$ structure. This result can be attributed to the decrease of the degree of sulfidation along with the sulfidation temperature. The sulfur species are gradually removed via the excessively high temperature, leading to the crystal reconstruction.[6] As shown in the Figure S19, the surface morphologies of prepared samples were investigated using SEM measurement. Most of the samples well-maintained the unique inverse opal structure even after the thermal sulfidation process. However, the IO-$Ni_xCo_{9-x}S_8$@NSC prepared at 600°C showed structural sollapse due to the excessive thermal energy and self aggregation phenomenon. In the Figure S20, the LSV polarization curves for OER and ORR demonstrate that the IO-$Ni_xCo_{9-x}S_8$@NSC-550 sample has the highest electrocatalytic activity among the prepared samples. This result suggests that the thermal sulfidation at 550°C not only preserves the uniform porous structure but generate highly active Ni-doped Co_9S_8 species without any phase impurity. Consequently, the optimal sulfidation temperature was concluded to be 550°C for utilization of electrocatalyst as air cathode for rechargeable Zn-air battery.

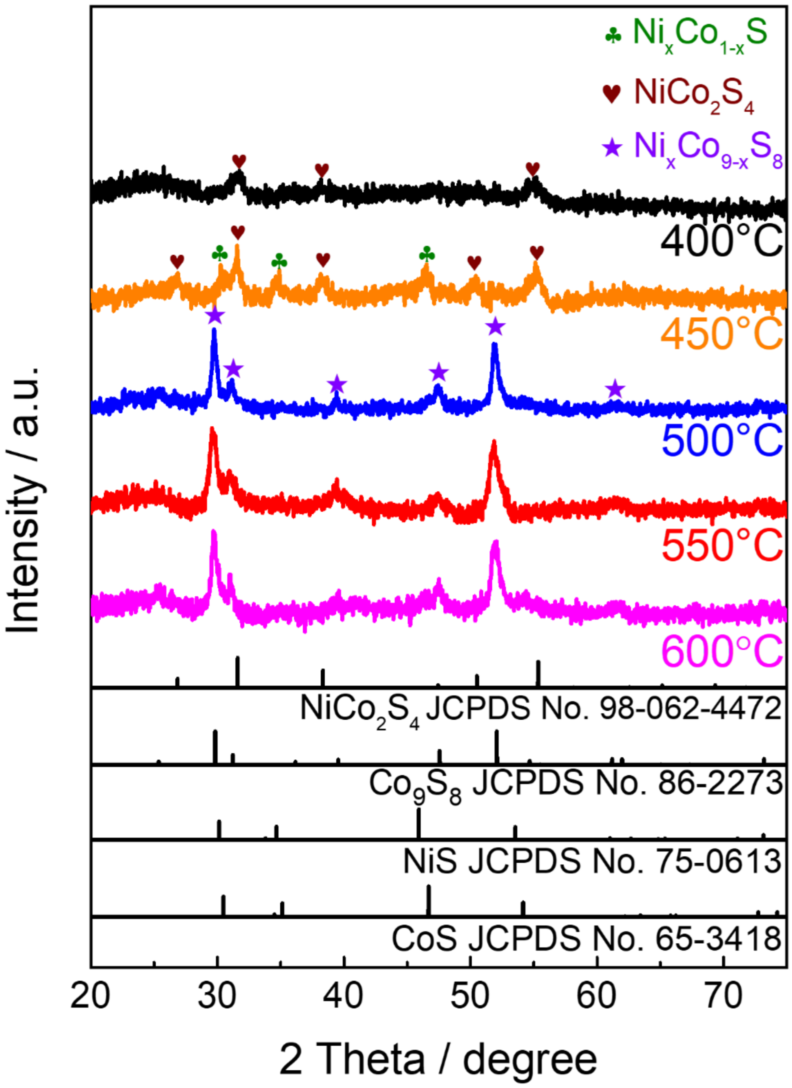

Figure S18. X-ray diffraction (XRD) patterns of IO-$Ni_xCo_{9-x}S_8$@NSC samples prepared with various sulfidation temperatures.

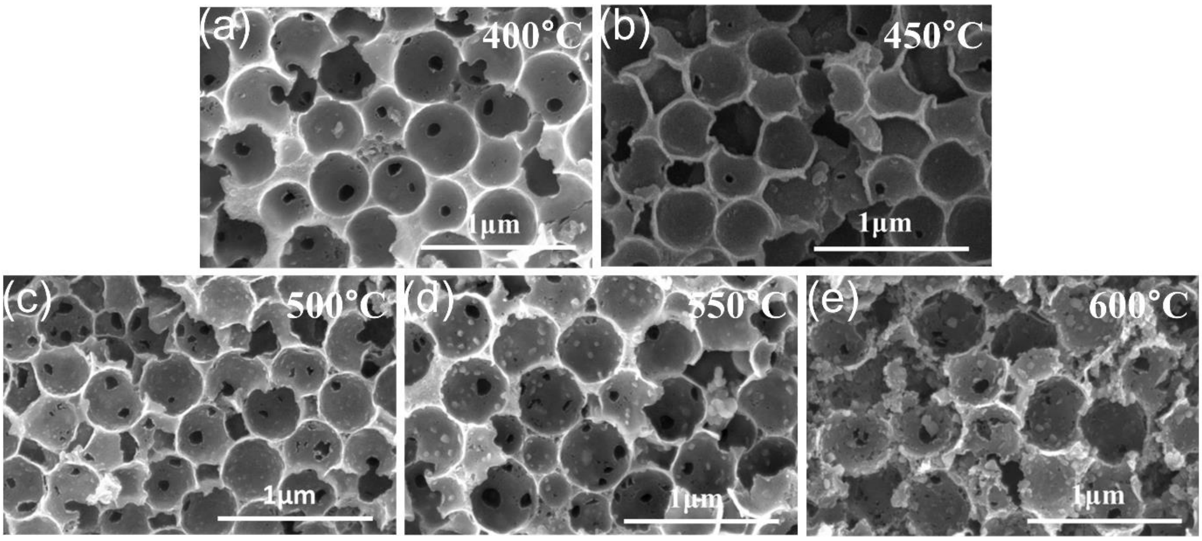

Figure S19. Scanning electron microscopy (SEM) images of IO-$Ni_xCo_{9-x}S_8$@NSC samples prepared with various sulfidation temperatures of (a) 400°C, (b) 450°C, (c) 500 °C, (d) 550 °C, and (e) 600 °C.

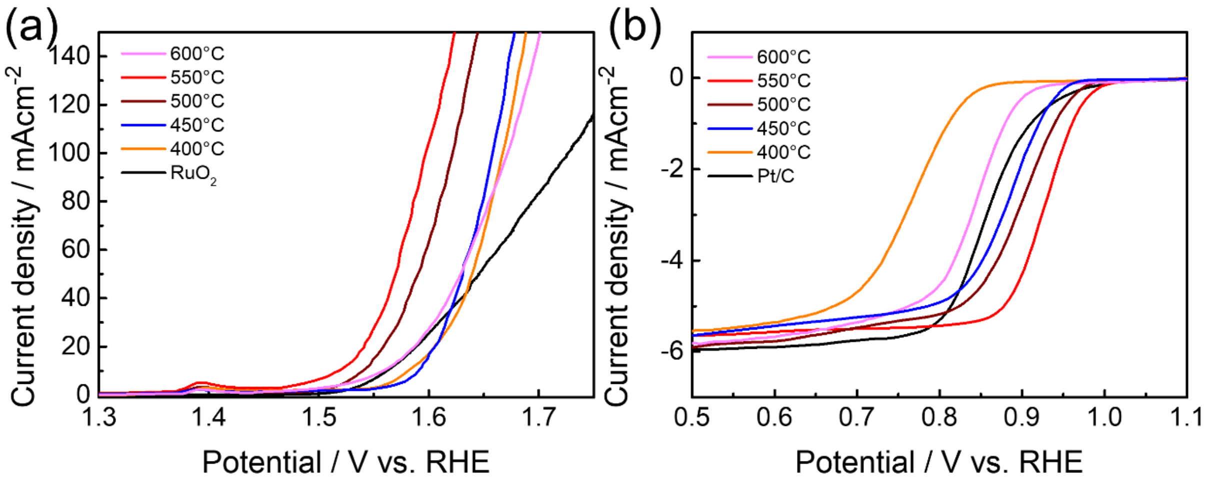

Figure S20. Polarization curves of IO-$Ni_xCo_{9-x}S_8$@NSC samples prepared with various annealing temperatures (a) in O_2-saturated 0.1 M KOH solution and (b) N_2-saturated 0.1 M KOH solution to measure electrochemical activity toward ORR and OER, respectively.

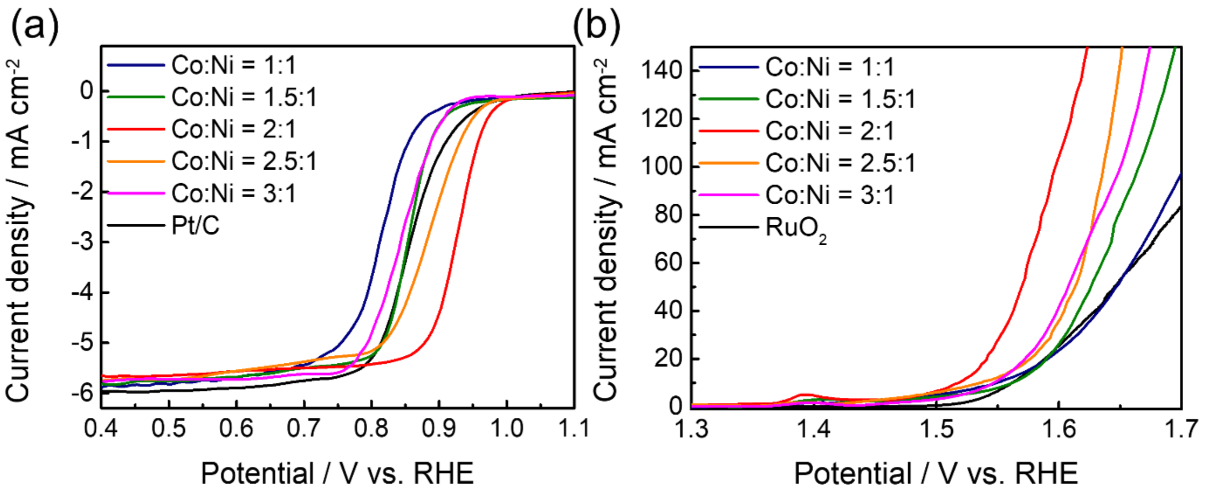

Figure S21. LSV Polarization curves of IO-$Ni_xCo_{9-x}S_8$@NSC samples prepared with different molar ratios of Co and Ni (Co:Ni = 1:1, 1.5:1, 2:1, 2.5:1 and 3.5:1) (a) in O_2-saturated and (b) N_2-saturated 0.1 M KOH solution to measure electrochemical activity toward ORR and OER, respectively.

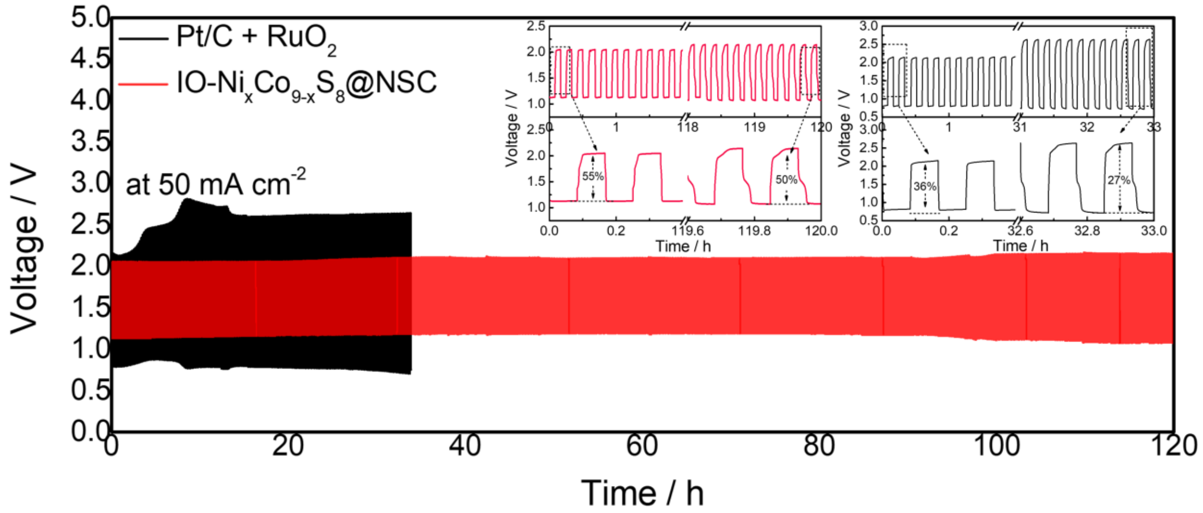

Figure S22. Long-term galvanostatic charge/discharge process at a pulse current density of 50 mA cm^{-2} with 5 min intervals.

Physicochemical analysis after the cyclability test

To further confirm the physicochemical characteristics of IO-$Ni_xCo_{9-x}S_8$@NSC after the cyclability test, we collected the IO-$Ni_xCo_{9-x}S_8$@NSC-coated GDL electrode following the long-term charge/discharge process of ZAB over 960 cycles. SEM was employed to examine the surface morphology and structural characteristics, as illustrated in Figure S23. The SEM image reveals that, despite the extended cyclability test under harsh alkaline conditions, the well-defined inverse opal 3D structure remains intact, demonstrating the excellent structural stability of the prepared electrocatalyst. Following the ZAB cycling test, XRD analysis was also conducted using the IO-$Ni_xCo_{9-x}S_8$@NSC-coated GDL electrode (Figure S24a). In the post-sample, the diffraction peaks for cubic-structured Co_9S_8 were clearly observed with graphite carbon peaks, which is originated by GDL substrate. Although the XRD peak intensities were significantly decreased, no other impurity peaks are observed after the cycling test. The slight decrease in the peak intensities for Co_9S_8 can be attributed to the surface amorphization induced by the charge/discharge process under alkaline condition.[7] To further examine the surface amorphization phenomenon, Raman spectroscopy was employed to analyze the chemical bonding characteristics of post-sample (Figure S24b). Before the cycing test, no notable peaks were observed within the Raman shift range of 400–1200 cm^{-1}. However, following the cycling test, the characteristic peaks at 554 and 650 cm^{-1} newly appeared, which are ascribed to the stretching vibrations of CoOOH. Furthermore, the strong peak at 472 cm^{-1} and 1150 cm^{-1} are attributed to the NiOOH and O−O vibration, respectively, demonstrating that the surface amorphization of $Ni_xCo_{9-x}S_8$ generates the metal oxyhydroxide species.[1-4] As proved in the previous works, electrocatalyst could be reconfigured into active metal oxyhydroxides (MOOH) during the OER process. In general, the MOOH is knwon as highly active species for oxygen electrocatalysis. Therefore, the *in-situ* surface reconstruction also

contribute to enhancing the electrocatalytic performance of rechargeable ZAB during the long-term operation.[5]

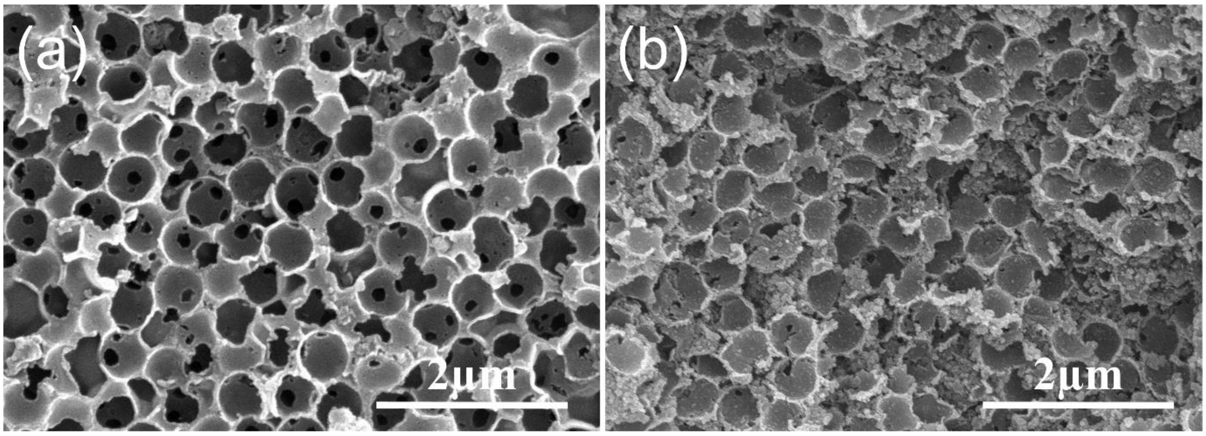

Figure S23. Scanning electron microscopy (SEM) of IO-$Ni_xCo_{9-x}S_8$@NSC electrocatalyst (a) before and (b) after the long-term galvanostatic charge/discharge process of ZAB over 960 cycles.

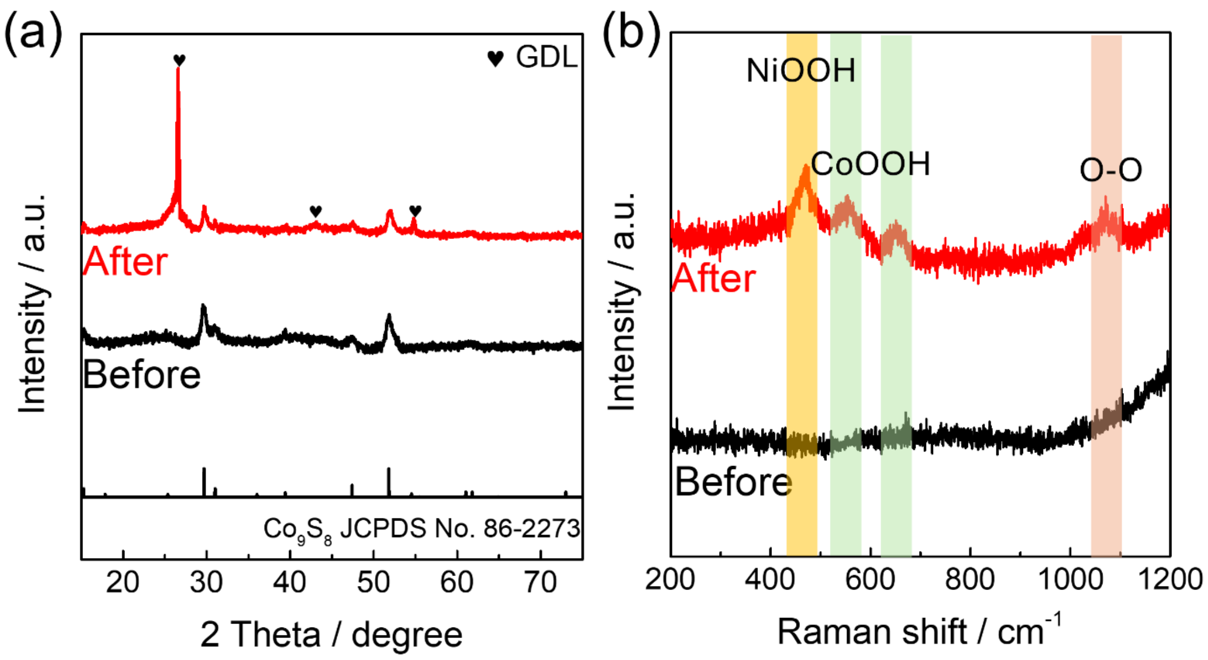

Figure S24. (a) X-ray diffraction (XRD) patterns and (b) Raman spectra of IO-$Ni_xCo_{9-x}S_8$@NSC before and after ZAB cyclability test.

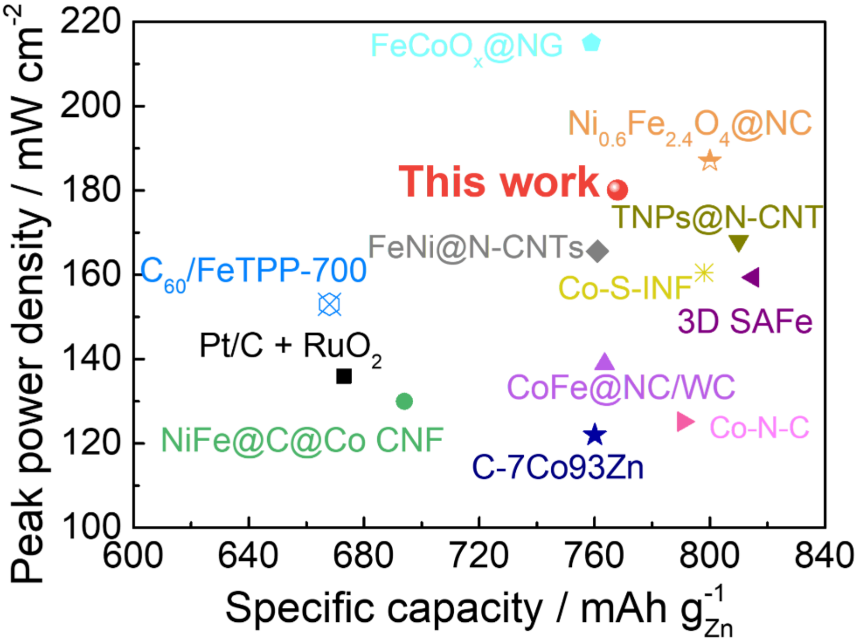

Figure S25. Comparison of battery performance of IO-$Ni_xCo_{9-x}S_8$@NSC-ZAB with previously reported bifunctional electrocatalyst-based ZABs.

Table S1. The elemental proportion of Ni, Co, N, S, and C for IO-$Ni_xCo_{9-x}S_8$@NSC determined by TEM-EDS, XPS, ICP-OES, and EA measurement

	TEM-EDS	XPS survey	ICP-OES	EA
	**IO-$Ni_xCo_{9-x}S_8$@NSC**			
Ni (at%)	2.21	2.49	34.11	-
Co (at%)	3.70	5.03	65.89	-
N (at%)	15.81	16.15	-	16.20
S (at%)	7.71	9.91	-	8.52
C (at%)	71.11	67.14	-	75.28
Total (at%)	100	100	100-	100

Table S2. ORR/OER activities of as-prepared catalysts. The thermodynamic equilibrium potential for oxygen electrocatalysis (E_{eq} (OH^-/O_2) = 1.23 V) is adopted as a reference to reflect each overpotential regarding ORR and OER

**Catalysts**	**ORR**		**OER**	ΔE /V (=$\mathbf{E_{j=10}^{B} – E_{1/2}^{A}}$)
	$\mathbf{E_{1/2}^{A}}$ **/ V**	$\boldsymbol{j_{L,\ ORR}}$ **/ mA cm^{-2} at 0.4V**	$\mathbf{E_{j=10}^{B}}$ **/ V at 10mA cm^{-2}**	
IO-$Ni_xCo_{9x}S_8$@NSC	0.926	5.70	1.519	0.593
NiCo@NC	0.832	-5.35	1.622	0.79
B-$Ni_xCo_{9-x}S_8$@NSC	0.740	4.92	1.584	0.844
IO-NiS@NSC	0.778	5.74	1.603	0.825
IO-CoS@NSC	0.808	4.29	1.627	0.819
Pt/C + RuO_2	0.858	5.96	1.696	0.838

Table S3. Comparison of electrocatalytic ORR performance of IO-$Ni_xCo_{9-x}S_8$@NSC with previously reported transition metal-based materials.

**Samples**	**Half wave-Potential (V vs. RHE)**	**Tafel slope (mV dec^{-1})**	**References**
IO-$Ni_xCo_{9-x}S_8$@NSC	0.926	44.1	**This work**
CoP/CoO@MNC-CNT	0.838	70.15	*Small* **2023**, *19*, 2206341
Co_2P/Co-NC	0.88	70	*ACS Appl. Mater. Interfaces* **2023**, *15*, 9240
Se-doped MOF CoS_2 HSs	0.88	49.8	*Appl. Catal. B-Environ* **2023**, *330*, 122523
Co_9S_8@Co/Mn-S,N-PC	0.85	85.5	*J. Colloid Interface Sci.* **2022**, *608*, 2100
CoNi/Ti_4O_7@NS-CNFs	0.86	55.1	*J. Colloid Interface Sci.* **2023**, *630*, 763
Co/CoS_2/NSCNT	0.89	31.8	*J. Energy Storage* **2024,** *86* 111298
Fe-Se/NC	0.925	56	*Angew. Chem.-Int. Edit.* **2023**, *62*, e202219191
FeCo@NMC	0.90	51.9	*J. Alloys Compd* **2023**, *935*, 168107
NCMO@rGO	0.78	48	*Adv. Sci*, **2023**, *10*, 2303525
NPC-950	0.88	63.7	*Adv. Funct. Mater*, ***2024***, *34*, 2314444

Table S4. Comparison of electrocatalytic OER performance of IO-$Ni_xCo_{9-x}S_8$@NSC with previously reported transition metal-based materials.

**Samples**	***η* (mV) at *j* = 10 mA cm^{-2}**	**Tafel slope (mV dec^{-1})**	**References**
IO-$Ni_xCo_{9-x}S_8$@NSC	289	75.79	**This work**
Co_4N@CoON/PCGN	360	63	*Adv. Mater.* **2024**, *36*, 2311105
CoFe-S@3D-S-NCNT	310	67	*Small* **2023**, *19*, 2206067
Fe/Co_9S_8@NSC	332	117	*Electrochim. Acta* **2024**, *476*, 143767
J−CeO_2/ZCS	290	55	*Appl. Catal. B-Environ.* **2024**, *342*, 123459
Co/Co_9S_8-NMC	298	31.69	*Mater. Today Energy* **2023**, *37*, 101398
Co/CoO/Co_3O_4/NCS	285	73.9	*Chem. Eng. J.* **2023**, *453*, 139831
Ce-Co_3O_4	340	105	*Adv. Funct. Mater.* **2023**, *33*, 2212021
NiFe-MOF/$NiFe_2O_4$	302	93	*J. Alloys Compd.* **2023**, *943*, 169144
Cu,Co/NSC2	339	75.9	*Adv. Funct. Mater.* **2024**, *34*, 2311664
$Fe_{3\%}Co_{3\%}Ni_{9\%}$-NC1000	330	90.7	*Appl. Catal., B-Environ.* **2024**, *342*, 123438

Table S5. Comparison of the electrocatalytic performance of rechargeable Zn-Air batteries assembled with various electrocatalysts.

**Catalysts**	**Peak power density (mW cm^{-2})**	**Specific capacity (mAh g_{Zn}^{-1})**	**References**
IO-$Ni_xCo_{9-x}S_8$@NSC	180.2	768	**This work**
FeNi@N−CNTs	165.6	761	*ChemCatChem* **2024**, *16*, e202301195
NiFe@C@Co CNFs	130	694	*Small* **2022**, *18*, 2200578
CoFe@NC/WC	138.9	763.5	*Energy Environ. Mater*. **2024**, *7*, e12499
TNPs@N-CNT	90	803	*Carbon* **2024**, *220*, 118859
C-7Co93Zn	122	760.1	*Mater. Today Energy* **2022**, *24*, 100935
3D SAFe	156	815	*Nano Lett.* **2022**, *22*, 7836
Co-N-C	125.2	790.8	*Nano Res.* **2022**, *15*, 7959
$FeCoO_x$@NG	215	758.9	*Carbon Energy* **2023**, *5*, e274
C_{60}/FeTPP-700	153	668	*Chin. Chem. Lett.* **2023**, *34*, 107601
$Ni_{0.6}Fe_{2.4}O_4$@NC	187.1	800	*ACS Appl. Mater. Interfaces* **2024**, *16*, 11537
Co-S-INF	160.5	778.9	*Nanoscale* **2024**, *16*, 4710

References

[1] E. Budiyanto, S. Salamon, Y. Wang, H. Wende, H. Tüysüz, *JACS Au* **2022**, *2*, 697.

[2] Y. Xie, L. Sun, X. Pan, Z. Zhou, G. Zhao, *Appl. Catal. B Environ.* **2023**, *338*, 123068.

[3] Z. Zhou, Y. nan Xie, L. Sun, Z. Wang, W. Wang, L. Jiang, X. Tao, L. Li, X. H. Li, G. Zhao, *Appl. Catal. B Environ.* **2022**, *305*, 121072.

[4] P. W. Menezes, S. Yao, R. Beltrán-Suito, J. N. Hausmann, P. V. Menezes, M. Driess, *Angew. Chem. Int. Ed.* **2021**, *133*, 4690.

[5] Y. P. Deng, Y. Jiang, R. Liang, S. J. Zhang, D. Luo, Y. Hu, X. Wang, J. T. Li, A. Yu, Z. Chen, *Nat. Commun.* **2020**, *11*, 1952.

[6] Q. Ren, Z. Wu, S. Hu, L. He, S. Su, Y. Wang, L. Jiang, J. Xiang, *Sci. Total Environ.* **2020**, *741*, 140193.

[7] T. Zhang, X. Zhu, D. D. Ye, R. Chen, Y. Zhou, Q. Liao, *Nanoscale* **2020**, *12*, 20270.
